# Supplementary material for: Hydrogen isotope labeling unravels origin of soil-bound organic contaminant residues in biodegradability testing
Source: Nat Commun. 2024 Oct 24;15:9178. doi: 10.1038/s41467-024-53478-w (PMC11502848; doi:10.1038/s41467-024-53478-w)
Supplement: Supplementary file 1 — Supplementary Information [file 41467_2024_53478_MOESM1_ESM.pdf]

Supplementary Information (SI) for

Hydrogen isotope labeling unravels origin of soil-bound organic contaminant residues in biodegradability testing

Sophie Lennartz [1,2], Harriet A. Byrne [1,3], Steffen Kümmel [4], Martin Krauss [5], Karolina M. Nowak [4,6,*]

[1)] Department of Molecular Environmental Biotechnology, Helmholtz-Center for Environmental Research – UFZ, Permoserstr. 15, 04318 Leipzig, Germany

[2)] Department of Environmental Science, Aarhus University, Frederiksborgvej 399, 4000 Roskilde, Denmark.

[3)] Department of Environmental Analytical Chemistry, Helmholtz-Center for Environmental Research – UFZ, Permoserstr. 15, 04318 Leipzig, Germany

[4)] Department of Technical Biogeochemistry, Helmholtz-Center for Environmental Research – UFZ, Permoserstr. 15, 04318 Leipzig, Germany

[5)] Department of Exposure Science, Helmholtz-Center for Environmental Research – UFZ, Permoserstr. 15, 04318 Leipzig, Germany

[6)] Division of Geobiotechnology, Institute of Biotechnology, Technische Universität Berlin, Ackerstraße 76, 13355 Berlin, Germany

* corresponding author: karolina.nowak@ufz.de

This SI file includes:

Supplementary Note 1 to **7**

Supplementary Method 1 to **4**

Supplementary Figure 1 to **10**

Supplementary Table 1 to **8**

Supplementary Notes:

Supplementary Note 1: Estimation of C and H supply for microbial synthesis of biomolecules.

Supplementary Note 2: Estimation of D loss from GLP and 2,4-D to water.

Supplementary Note 3: Extraction of desorbable 2,4-D, GLP and SMX from soil.

Supplementary Note 4: Statistical data analysis.

Supplementary Note 5: Calculation of $^{13}C_{biogenic\ NERs}$ using the microbial turnover to biomass (MTB) model.

Supplementary Note 6: Assessment of D-label stability in water.

Supplementary Note 7: Respiration of soil spiked with 2,4-D, GLP and SMX.

Supplementary Methods:

Supplementary Method 1: Detection limits for total NER and bioNER analysis using ^{13}C and D tracers.

Supplementary Method 2: Conditions for amino acids and isotope-labeled amino acids separation on gas chromatography-mass spectrometry (GC-MS) and gas chromatography-isotope ratio mass spectrometry (GC-IRMS).

Supplementary Method 3: Derivatization correction of AAs.

Supplementary Method 4: Calculation of ^{13}C- and D-label integration into total NERs and tAAs.

Supplementary Figures:

Supplementary Fig. 1: Loss of D from a D-labeled compound to soil water.

Supplementary Fig. 2: QQ-plots of normalized z-residuals for NERs of GLP in sterile soil fitted with one-way ANOVA.

Supplementary Fig. 3: QQ-plots of normalized z-residuals for NERs of GLP in biologically active soil fitted with one-way ANOVA.

Supplementary Fig. 4: QQ-plots of normalized z-residuals for tAAs in the GLP treatment in biologically active soil fitted with one-way ANOVA.

Supplementary Fig. 5: QQ-plots of normalized z-residuals for glycine in the GLP treatment in biologically active soil fitted with one-way ANOVA.

Supplementary Fig. 6: Respiration of soil incubated with (a): 2,4-dichlorophenoxyacetic acid (2,4-D), (b): glyphosate (GLP),and (c): sulfamethoxazole (SMX).

Supplementary Fig. 7: Release of D from C–D bonds of a D-labeled substrate (reactant RD_2) during its enzymatic cleavage.

Supplementary Fig. 8: Degradation pathways of glyphosate (GLP) labeled at position 2-C leading to the formation of labeled sarcosine and glycine.

Supplementary Fig. 9: Degradation pathways of glyphosate (GLP) labeled at position 3-C leading to the formation of labeled aminomethylphosphonic acid (AMPA) and labeled sarcosine.

Supplementary Fig. 10: Contribution of the amino acid glycine to the total measured amino acid (tAA) pool for glyphosate (GLP) labeled at position 2 (a) or position 3 (b) with ^{13}C or D.

Supplementary Tables:

Supplementary Table 1: Estimated differences in the maximum amounts of microbial biomolecules synthesized in C- vs. H-limiting conditions.

Supplementary Table 2a: Contents of extractable residues (ERs) and non-extractable residues (NERs) measured by EA-IRMS, and total isotope label recovery on day 0 for 2,4-D, GLP and SMX.

Supplementary Table 2b: Contents of extractable residues (ERs) measured by GC-MS (2,4-D) or LC-MS (GLP, SMX) and non-extractable residues (NERs) by EA-IRMS, and their total isotope label recoveries on day 0.

Supplementary Table 3: P-values of Welch tests and adjusted values (p_{adj}) after Holm-Bonferroni correction for multiple testing for 2,4-D and SMX.

Supplementary Table 4: Cumulative endpoint mineralization of $^{13}C_{6\text{-}2,4\text{-}D}$ (day 36), 2-$^{13}C_{GLP}$ (day 38), 3-$^{13}C_{GLP}$ (day 38) and $^{13}C_{6\text{-}SMX}$ (day 72) used for MTB calculations.

Supplementary Table 5: Values of Gibbs energy of formation.

Supplementary Table 6: Input parameters and results of MTB modelling.

Supplementary Table 7: Stability assessment of the test compounds in aqueous solution after 6-day incubation at 20°C.

Supplementary Table 8a: Estimated detection limits for $^{13}C_{NERs}$.

Supplementary Table 8b: Estimated detection limits for D_{NERs}.

Supplementary Table 8c: Estimated detection limits for $^{13}C_{AAs}$ and D_{AAs} for GLP.

Supplementary Table 8d: Estimated detection limits for $^{13}C_{AAs}$ and D_{AAs} for 2,4-D.

Supplementary Notes

Supplementary Note 1: Estimation of C and H supply for microbial synthesis of biomolecules

To estimate quantitatively how much lower the incorporation of H vs. C tracers into microbial biomass may be, we calculated the theoretical maximum amounts of different biomolecules that could be produced based on the total 'supply' of C and H in our model soil. For simplicity, the total C or H present in soil was assumed to be used for biosynthesis and other limiting factors besides the abundance of the atomic building blocks were neglected. The total C in the system (C_{tot}) was estimated as the sum of the C content in NERs (C_{NERs}) and extractable residues (C_{ERs}) measured by elemental analyzer-combustion-isotope ratio mass spectrometry (EA-C-IRMS) on day 0 according to **Supplementary Equation 1**. Averaged for 2,4-dichlorophenoxy acetic acid (2,4-D), glyphosate (GLP) and sulfamethoxazole (SMX) in biotic treatments, the C_{tot} amounted to 1.9 mmol C (g d.w.)$^{-1}$ equivalent to 2.4 mass%.

$$C_{tot} = C_{NERs} + C_{ERs} = 1.87 + 0.03 = 1.90 \text{ mmol C (g d.w.)}^{-1} \qquad \textbf{(1)}$$

The total H content (H_{tot}) stems from both H in soil water ($H_{soil\ water}$) and the soil-bound H (H_{NERs}). Average H_{NER} contents in this study amounted to 4.0 mmol H g d.w.$^{-1}$ in the biotic treatments. $H_{soil\ water}$ was calculated from the water content at 60% maximum water holding capacity (WHC_{max}) and the molar mass of H_2O according to **Supplementary Equation 2**. Hence:

$$H_{soil\ water} = 0.6 \times WHC_{max} \times (18\ [\text{g mol}^{-1}])^{-1} \times (2\ [\text{mol H/ mol } H_2O]) = 25 \text{ mmol H (g d.w.)}^{-1} \qquad \textbf{(2)}$$

$$H_{tot} = H_{NERs} + H_{soil\ water} = 4.0 + 25 = 29 \text{ mmol H (g d.w.)}^{-1} \qquad (3)$$

As shown in **Supplementary Table 1**, the estimated total H supply required to build different biomolecules was 6-11 times larger than the respective C supply. Consequently, biosynthesis is much more likely to be C- than H-limited, supporting our hypothesis of stronger C retention in microbial biomass. The exact differences between substrate-C and substrate-H utilization, however, depend on various other factors such as the molecular structure of the substrate (toxicity and energy required for breakdown), the microbial community composition (e.g. abundance of autotrophs vs. heterotrophs), and the soil properties (texture, WHC, etc.).

Supplementary Table 1: Estimated differences in the maximum amounts of microbial biomolecules synthesized in C- vs. H-limiting conditions. Estimates are based on the total measured C and H supply in the sandy loam used as model soil.

**Biomolecule**	**Sum formula**	**Demand per molecule**		**Maximum production per 1 g soil**		
		**C**	**H**	**C-limited (mmol)**	**H-limited (mmol)**	**Ratio H- vs. C-limited**
**Glucose**	$C_6H_{12}O_6$	6	12	0.316	2.384	7.5
**Alanine**	$C_3H_7NO_2$	3	7	0.633	4.086	6.5
**Citric acid**	$C_6H_8O_7$	6	8	0.316	3.575	11
**Acetic acid**	$C_2H_4O_2$	2	4	0.949	7.151	7.5
**Pyruvic acid**	$C_3H_4O_3$	3	4	0.633	7.151	11
**Palmitic acid**	$C_{16}H_{32}O_2$	16	32	0.119	0.894	7.5
**Saturated fatty acid**	$CH_3(CH_2)_nCOOH$	2+n	4+2n	0.56+1.25n	7.25+14.5n	7.5

Supplementary Note 2: Estimation of D loss from GLP and 2,4-D to water

The maximum D loss from GLP and 2,4-D to water and the resulting D_2O concentration (% $D_2O/(D_2O+H_2O)$) was estimated based on the known biodegradation pathways for the two compounds as shown below (**Supplementary Equations 4-9**) [1,2]. For simplicity, it was assumed that all D bound in the compounds is released first as H_2O before losing light ^1H, i.e. 2D for $D_{2\text{-GLP}}$ and 3D for $D_{3\text{-2,4-D}}$. Therefore, the estimated D_2O loss amounted to 1 and 1.5 molecules D_2O per molecule CO_2 produced. Based on the measured $^{13}CO_2$ evolution (data not shown) and water content of the model soil (12.5 mmol H_2O/g wet soil at 60% WHC_{max}), we then estimated the D_2O dilution. As a reference, in **Supplementary Fig. 1** the maximum D_2O content in D-depleted water used in our study (1ppm) is shown. The maximum expected D_2O concentration in soil water after GLP degradation via the aminomethylphosphonic acid (AMPA) pathway would be 0.00124 % or 12.4 ppm (including trace levels of D_2O potentially present in D-depleted water), which is over 10-fold lower than the natural D-abundance in the dry soil (0.0149 at%) or natural water (~0.015 at%). This shows that after D loss to water, only a very low fraction of ~0.001% of D may be taken up again for biosynthesis (**Supplementary Fig. 1**). Effects of isotopic fractionation are neglected in this simplified calculation as they could vary widely for different substrates, degraders and metabolic pathways [3].

2,4-D degradation (based on Trapp et al., 2022) [1]:

$$C_8H_6O_3Cl_2 + 7.5O_2 \leftrightharpoons 8CO_2\,(g) + 2H_2O\,(l) + 2HCl \quad (4)$$

GLP biodegradation (based on Brock et al, 2019) [2]

Sarcosine pathway:

$$C_3H_8NO_5P + H_2O\,(l) \leftrightharpoons C_3H_7NO_2 + H_3PO_4 \quad (5)$$

$$C_3H_7NO_2 + 0.5O_2 \leftrightharpoons C_2H_5NO_2 + CH_2O \quad (6)$$

$$C_2H_5NO_2 + CH_2O + 2.5O_2 + H^+ \leftrightharpoons 3CO_2\,(g) + 2H_2O\,(l) + NH_4^+ \quad (7)$$

$$C_3H_8NO_5P + CH_2O + 3O_2 + H^+ \leftrightharpoons 3CO_2\,(g) + 1H_2O\,(l) + NH_4^+ \quad (8)$$

AMPA pathway:

$$C_3H_8NO_5P + 1.5O_2 \leftrightharpoons CH_6NO_3P + 2CO_2\,(g) + H_2O\,(l) \quad (9)$$

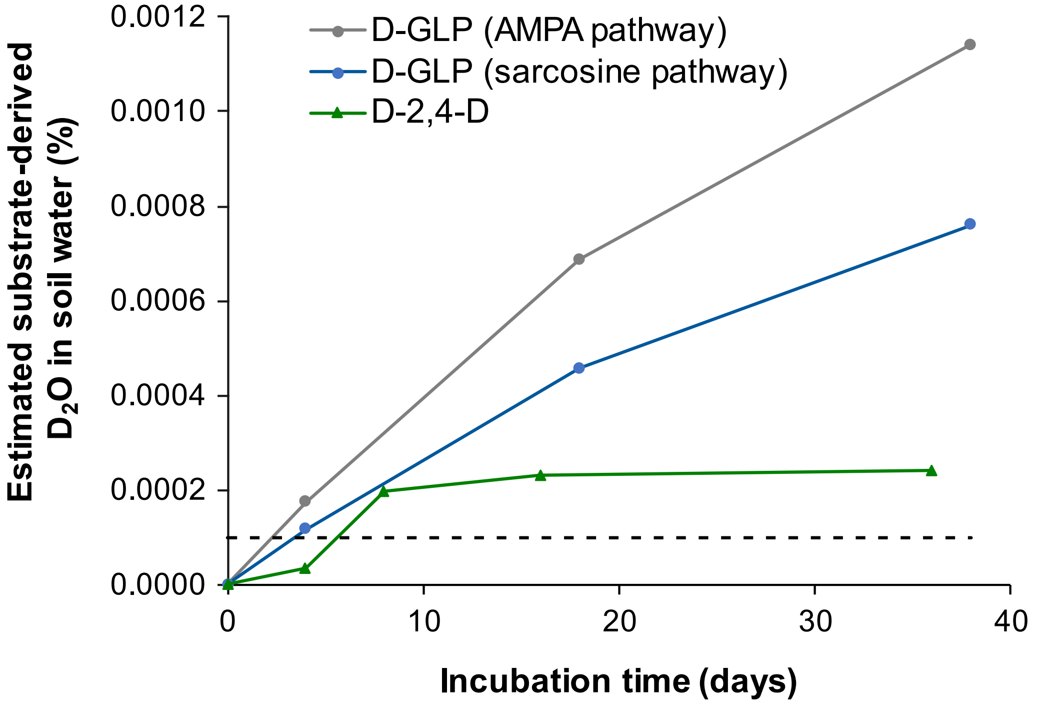

Supplementary Fig. 1: Loss of D from a D-labeled compound to soil water. **Green triangles**: D-labeled 2,4-dichlorophenoxyacetic acid (D-2,4-D); **grey dots**: D-labeled glyphosate (D-GLP) biodegraded via the aminomethylphosphonic acid (AMPA) pathway; **blue dots**: GLP biodegraded via the sarcosine pathway. The **black dotted line** shows the maximum background concentration of deuterated water (D_2O) in the soil water (0.0001%). The main increase in the loss of D from D-2,4-D to soil water occurred by day 8, whilst for GLP biodegraded via the AMPA and sarcosine pathway, loss continued until the end of the incubation period. The final estimated D loss to the soil water was lowest for D-2,4-D (0.00024%), followed by D-GLP biodegraded via the sarcosine pathway (0.00076%), and highest for D-GLP biodegraded via the AMPA pathway (0.00114%).

Supplementary Note 3: Extraction of desorbable 2,4-D, GLP and SMX from soil

Extraction of desorbable 2,4-D, GLP and SMX from soil was based on extraction protocols for the same reference soil previously developed in our lab. We performed recovery tests with the respective compound at the concentration to be spiked on day 0 of the incubation. For SMX, we chose the method which yielded the best recovery while being time-efficient (i.e. shortest extraction time after which no more compound could be released). We tested the recovery of the respective compound directly (maximum 20 min) after soil spiking in extraction efficiency tests as well as on day 0 of the main incubation experiment. Noteworthy is here that the preparation of soil batches during the main experiment took some time, so that the day 0 soil samples were frozen at -20°C about 2 hours after spiking and were analyzed at a later time after de-frosting. Therefore, recoveries of GLP and SMX on day 0 might have been lowered by sorption to the soil compared to recovery of these compounds directly after spiking. When feasible, we also measured the concentration of 2,4-D using gas chromatography mass spectrometry (GC-MS) after derivatization with methanol/trimethylchlorosilane [4]. GLP derivatized with Fluorenylmethoxycarbonyl-chloride (FMOC-Cl) and SMX were quantified using liquid chromatography mass spectrometry (LC-MS) [5]. When feasible, we also quantified the total ^{13}C and D isotope label in water and solvent extracts using EA-IRMS. For ^{13}C measurements a total extract volume of 1 mL was pipetted in small tin capsules (5 × 13 mm, HEKAtech) and evaporated to dryness prior to measurement. For D measurements, a small volume of 0.5 µL extract was collected in COM-AID™ (Leco Corporation, U.S.) and carefully sealed in the tin capsules.

The measurement of total D in water extracts by EA-IRMS showed highly variable results for all biotic soils (2,4-D, GLP and SMX) due to the high background D; thus, it might be not reliable in contrast to the measurements for abiotic experiments. The D-water measurement was needed for quantification of the mineralization of the D-compounds in biotic experiments. In abiotic experiments, we did not observe any mineralization; therefore, the D-water was evaporated prior to the EA-IRMS measurement, and this helped to obtain more reliable results. The quantitation of total D in borax extracts of all soils incubated with D_{GLP} using EA-IRMS was also impossible, even after the evaporation of aqueous borax.

2,4-D. For the recovery test, soil was spiked with 10 mg kg^{-1} of unlabeled 2,4-D dissolved in methanol. After spiking, the soil was kept in the fume hood for 15 min in order to evaporate the excess methanol. The 2,4-D was then extracted into a methanol/water (1:1; v:v) mixture on a horizontal shaker for 2 hours. After shaking, the soil extract was centrifuged (10 min, 5000 rpm) and the supernatant was collected into a separate flask before measuring the concentration of 2,4-D by GC-MS. The recovery of unlabeled 2,4-D was >98±5%. We also measured the recovery of $^{13}C_{6\text{-}2,4\text{-}D}$ and

$D_{3\text{-}2,4\text{-}D}$ in soil 2 hours after spiking in the main incubation experiment, which ranged between 73±16% and 79±18% when measured by EA-IRMS (see **Supplementary Table 2a**) and 92±6.5% and 101±5.1% using GC-MS (**Supplementary Table 2b**). These values are comparable to the 89±2.1% obtained by Girardi et al. [6] who used the same solvent mixture and accelerated solvent extraction.

GLP. Previous recovery tests performed by Muskus et al. [5] in our lab showed about 93%±3% recovery of unlabeled GLP and $^{13}C_3$,$^{15}N_{GLP}$ (measured by LC-MS/MS) for the same soil using a single extraction with 40 mM borate buffer followed by SPE-cleanup over Oasis HLB cartridges. In this study, prior to 40 mM borate buffer extraction, GLP was first extracted into 1 mL of water by shaking for 15 min on a vortex shaker to enhance the recovery. Thereafter, the sample was centrifuged at 3,500 rpm (Eppendorf 5424 R, Germany) and the same soil sample was subjected to extraction with 40 mM borate buffer (GLP) on a horizontal shaker for 15 min, followed by 15 min centrifugation at 6500 rpm (Sigma 3K18; Merck) and SPE-cleanup. The recovery of $^{13}C/D_{GLP}$ on day 0 (2 hours after spiking) was < 10% in water extracts, with the notable exception of 103±63% recovery for 2-C–$D_{2\text{-}GLP}$ measured by EA-IRMS. In the abiotic experiments between 83±6.1% and 87±6.5% of spiked GLP was measured in borate buffer by EA-IRMS (**Supplementary Table 2a**), and between 52±8.3% and 65±3.2% based on LC-MS measurements (**Supplementary Table 2b**). Recoveries were a bit lower in the biotic experiments (17±3.6 to 36%), possibly because of the longer sample preparation time. The recoveries were overall lower than the estimates in the recovery tests (93%±3%) by Muskus et al. [5], who performed the extraction with 40 mM borate buffer directly after spiking the soil with GLP. As GLP is known to quickly sorb to soil [7], this may have affected recovery in our study already within 2 hours after spiking.

SMX. Extraction of SMX ($^{13}C_{6\text{-}SMX}$, $D_{4\text{-}SMX}$) was based on an adaptation of the protocol by Gros et al. who had reached about 80% recovery [8]. In our protocol, 1 g of the reference soil was spiked with 20 mg kg^{-1} of unlabeled SMX dissolved in methanol. Similarly to the 2,4-D spiking procedure, the excess of methanol was evaporated under a fume hood for about 20 min before shaking with 4 mL dichloromethane (DCM) for 2 hours on a horizontal shaker. Our recovery tests with the soil spiked with unlabeled SMX dissolved in methanol showed a recovery up to maximum 80±10% (LC-MS/MS) using different solvent combinations – water, DCM, acetonitrile, shaking and ultrasonication. As expected most (90%) of the SMX was extracted into water, since SMX in the neutral range of pH 6.6 (our reference soil) displays high mobility ($LogK_{OW} < 1$) [9,10]. Any extended shaking or vortexing time did not increase SMX recovery. Based on the best recovery results obtained in screening tests, we extracted SMX from soil in two-steps – first with water (1 mL, 15 min, vortex) and then with DCM (DCM, 4 mL, 2 hours, horizontal shaking). The recovery of SMX in water

extracts ranged between 67±18% – 70%±63% by EA-IRMS and 60±4.5% – 78±17 % by LC-MS in the biotic experiment (**Supplementary Table 2a and 2b**). In the abiotic experiment, recoveries based on LC-MS were surprisingly about 2-fold higher (**Supplementary Table 2b**). The second extraction step with DCM yielded much lower recovery, up to 4.8±0.8%. Lower estimates of extractable SMX by EA-IRMS could be due to liquid losses during multiple pipetting and drying of extracts.

Supplementary Table 2a: Contents of extractable residues (ERs) and non-extractable residues (NERs) measured by EA-IRMS, and total isotope label recovery on day 0 for 2,4-D, GLP and SMX.

Compound	ERs-H_2O	ERs	NERs	Total
**Biotic incubations**				
$^{13}C_{6\text{-}2,4\text{-}D}$	n.a.	n.m.	n.d.	n.a.
$D_{3\text{-}2,4\text{-}D}$	n.a.	n.a.[1)]	1.6 ± 2.2	n.a.
2-$^{13}C_{\text{-}GLP}$	2.8 ± 1.1	23 ± 5.0	40 ± 3.1	66 ± 6.0
2-C–$D_{2\text{-}GLP}$	103 ± 63	n.a.[1)]	42 ± 3.2	*149 ± 63
3-$^{13}C_{\text{-}GLP}$	2.9 ± 1.1	17 ± 3.6	35 ± 6.4	54 ± 7.4
3-C–$D_{2\text{-}GLP}$	2.8 ± 18	n.a.[1)]	34 ± 6.5	*71 ± 19
$^{13}C_{6\text{-}SMX}$	67 ± 18	n.d.	60 ± 18	128 ± 25
$D_{4\text{-}SMX}$	70 ± 63	n.a.[1)]	26 ± 5.0	*100 ± 63
**Abiotic incubations**				
$^{13}C_{6\text{-}2,4\text{-}D}$	n.a.	73 ± 16	7.6 ± 2.0	80 ± 16
$D_{3\text{-}2,4\text{-}D}$	n.a.	79 ± 18	7.0 ± 3.9	86 ± 18
2-$^{13}C_{\text{-}GLP}$	4.6 ± 0.3	83 ± 6.1	18 ± 3.1	106 ± 6.9
2-C–$D_{2\text{-}GLP}$	9.5 ± 0.7	n.a.[1)]	22 ± 4.0	*90 ± 4.1
3-$^{13}C_{\text{-}GLP}$	5.0 ± 2.4	87 ± 6.5	30 ± 5.8	122 ± 9.0
3-C–$D_{2\text{-}GLP}$	5.9 ± 2.8	n.a.[1)]	28 ± 7.2	*86 ± 11
$^{13}C_{6\text{-}SMX}$	52 ± 11	4.8 ± 0.8	39 ± 6.0	95 ± 13
$D_{4\text{-}SMX}$	81 ± 25	0.13 ± 0.00	32 ± 4.9	113 ± 26

n.a.: not applicable, n.d.: not detectable. n.m: not measured, *total recovery including ERs (LC-MS) in **Supplementary Table .2b**, [1)] D in ERs not quantified due to too high D background in extraction solvent

Supplementary Table 2b: Contents of extractable residues (ERs) measured by GC-MS (2,4-D) or LC-MS (GLP, SMX), and non-extractable residues (NERs) by EA-IRMS, and their total isotope label recoveries on day 0.

Compound	ERs-H_2O	ERs	NERs	Total
		**Biotic incubations**		
**$^{13}C_{6\text{-}2,4\text{-}D}$**	n.a.	92 ± 6.5	n.d.	92 ± 6.5
**$D_{3\text{-}2,4\text{-}D}$**	n.a.	97 ± 3.7	1.6 ± 2.2	99 ± 4.3
**2-$^{13}C_{\text{-}GLP}$**	5.5 ± 0.7	25 ± 2.7	40 ± 3.1	71 ± 4.2
**2-C–$D_{2\text{-}GLP}$**	7.1 [1)]	29 [1)]	42 ± 3.2	82 ± 3.2
**3-$^{13}C_{\text{-}GLP}$**	4.4 ± 0.3	24 ± 2.6	35 ± 6.4	63 ± 7.0
**3-C–$D_{2\text{-}GLP}$**	7.8 [1)]	36 [1)]	34 ± 6.5	84 ± 6.5
**$^{13}C_{6\text{-}SMX}$**	78 ± 17	n.d.	60 ± 18	138 ± 24
**$D_{4\text{-}SMX}$**	60 ± 4.5	3.9 ± 1.2	26 ± 5.0	90 ± 6.8
		**Abiotic incubations**		
**$^{13}C_{6\text{-}2,4\text{-}D}$**	n.a.	96 ± 3.3	7.6 ± 2.0	104 ± 3.8
**$D_{3\text{-}2,4\text{-}D}$**	n.a.	101 ± 5.1	7.0 ± 3.9	108 ± 6.4
**2-$^{13}C_{\text{-}GLP}$**	6.8 ± 0.5	54 ± 4.1	18 ± 3.1	79 ± 5.2
**2-C–$D_{2\text{-}GLP}$**	7.1 ± 5.4	58 ± 0.6	22 ± 4.0	87 ± 6.7
**3-$^{13}C_{\text{-}GLP}$**	9.6 ± 0.2	65 ± 3.2	30 ± 5.8	104 ± 6.6
**3-C–$D_{2\text{-}GLP}$**	6.5 ± 6.1	52 ± 8.3	28 ± 7.2	87 ± 12
**$^{13}C_{6\text{-}SMX}$**	103 ± 1.6	0.15 ± 0.00	39 ± 6.0	142 ± 6.2
**$D_{4\text{-}SMX}$**	98 ± 1.9	0.16 ± 0.00	32 ± 4.9	130 ± 5.3

n.a.: not applicable, n.d.: not detectable, [1)] only one replicate analyzed

Supplementary Note 4: Statistical data analysis

Due to the very small sample size, homoscedasticity was assessed based on ratios of highest to lowest group variance. Variance ratios were > 3 in all cases, and hence unequal variances were assumed for all analyses. For 2,4-D and SMX Welch tests were used despite the small sample size preventing a proper assessment the underlying distributions as the Welch test was shown to be robust against deviations from normality[11]. For GLP, normality was assessed by visual inspection of quantile-quantile (QQ) plots of the groupwise normalized z-residuals to account for variance heterogeneity. QQ plots for NERs and tAAs showed consistent s-shaped patterns with data gaps and strong deviations from the straight line, suggesting light-tailed, non-normal distributions. We therefore chose non-parametric models for statistical analyses for GLP. Because of the small sample sizes, the applied tests may have been underpowered to detect very small differences, especially when only two valid measurements could be obtained per group. For statistical analyses and visualizations the following R packages were used: car[12], conover.test[13], rcompanion[14], ggpubr[15] and ggplot2[16].

4.1. GLP

4.1.1 Abiotic NERs

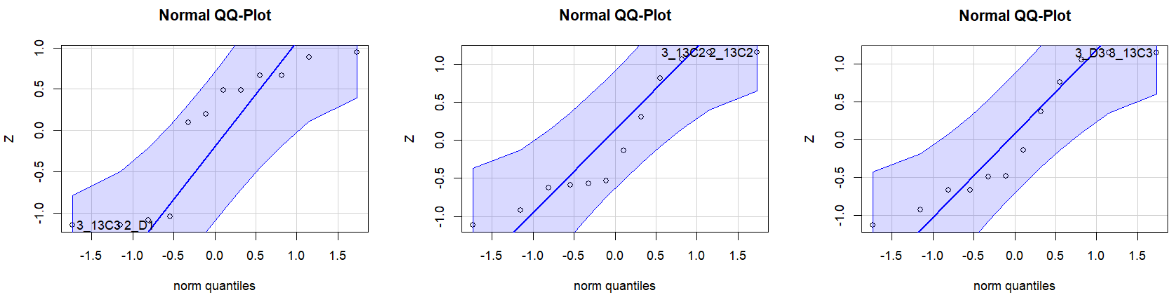

Supplementary Fig. 2: QQ-plots of normalized z-residuals for NERs of GLP in sterile soil fitted with one-way ANOVA. Open dots show the normalized residuals of the fitted anova model for day 0 (left), day 18 (middle) and day 36 (right). Blue lines and the blue shaded areas indicate the reference for a theoretical normal distribution with 95% confidence interval. The two data points with the largest deviation from a theoretical normal distribution are labeled. norm quantiles: quantiles normalized to the standard normal distribution (mean = 0, standard deviation = 1), z: z-residuals standardized to the standard normal distribution.

> kruskal.test(`Day 0` ~ Label, data = GLP_NERs_abiotic)

Kruskal-Wallis rank sum test

data: `Day 0` by Label

Kruskal-Wallis chi-squared = 8.7436, df = 3, **p-value = 0.0329**

data: `Day 18` by Label

Kruskal-Wallis chi-squared = 9.6667, df = 3, **p-value = 0.02162**

data: `Day 36` by Label

Kruskal-Wallis chi-squared = 9.4615, df = 3, **p-value = 0.02374**

Post-hoc comparison:
conover.test(GLP_NERs_abiotic$`day 0`, GLP_NERs_biotic$`Label, method="holm", list=TRUE, altp=TRUE)

Day 0:

Pairwise comparisons	t statistic	(Holm adjusted p-value)
2_13C - 2_D	-2.132007	(0.1312)
2_13C - 3_13C	-4.903616	(0.0071)*
2_D - 3_13C	-2.771609	(0.0969)
2_13C - 3_D	-4.477215	(0.0103)*
2_D - 3_D	-2.345207	(0.1411)
3_13C - 3_D	0.426401	(0.6811)

Day 18:

Pairwise comparisons	t statistic	(Holm adjusted p-value)
2_13C - 2_D	-1.386750	(0.2029)
2_13C - 3_13C	-6.933752	(0.0007)*
2_D - 3_13C	-5.547001	(0.0027)*
2_13C - 3_D	-4.437601	(0.0087)*
2_D - 3_D	-3.050851	(0.0474)*
3_13C - 3_D	2.496150	(0.0743)

Day 36:

Pairwise comparisons	t statistic	(Holm adjusted p-value)
2_13C - 2_D	0.774596	(0.4609)
2_13C - 3_13C	-5.422176	(0.0031)*
2_D - 3_13C	-6.196773	(0.0016)*
2_13C - 3_D	-3.098386	(0.0441)*
2_D - 3_D	-3.872983	(0.0189)*
3_13C - 3_D	2.323790	(0.0973)

4.1.2 Biotic NERs

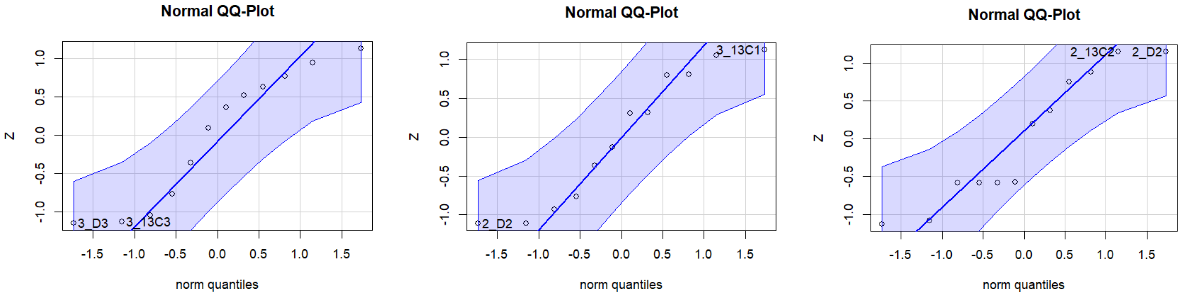

Supplementary Fig. 3: QQ-plots of normalized z-residuals for NERs of GLP in biologically active soil fitted with one-way ANOVA. Open dots show the normalized residuals of the fitted anova model for day 4 (left), day 18 (middle) and day 38 (right). Blue lines and the blue shaded areas indicate the reference for a normal distribution with 95% confidence interval. The two data points with the largest deviation from a theoretical normal distribution are labeled. norm quantiles: quantiles normalized to the standard normal distribution (mean = 0, standard deviation = 1), z: z-residuals standardized to the standard normal distribution.

> kruskal.test(`day 4` ~ Label, data = GLP_NERs_biotic)

Kruskal-Wallis rank sum test
data: `day 4` by Label
Kruskal-Wallis chi-squared = 10.385, df = 3, p-value = **0.01556**
data: `day 18` by Label
Kruskal-Wallis chi-squared = 10.385, df = 3, p-value = **0.01556**

data: `day 38` by Label

Kruskal-Wallis chi-squared = 10.421, df = 3, p-value = **0.01531**

Post-hoc comparison:

```
conover.test(GLP_NERs_biotic$`day 4`, GLP_NERs_biotic$`Label, method="holm", list=TRUE, altp=TRUE)
```

Day 4:

Pairwise comparisons	t statistic	(Holm adjusted p-value)
2_13C - 2_D	11.02270	(4.1e-06)*
2_13C - 3_13C	3.674234	(0.0188)*
2_D - 3_13C	-7.348469	(0.0004)*
2_13C - 3_D	7.348469	(0.0003)*
2_D - 3_D	-3.674234	(0.0125)*
3_13C - 3_D	3.674234	(0.0063)*

Day 18:

Pairwise comparisons	t statistic	(Holm adjusted p-value)
2_13C - 2_D	11.02270	(4.1e-06)*
2_13C - 3_13C	3.674234	(0.0188)*
2_D - 3_13C	-7.348469	(0.0004)*
2_13C - 3_D	7.348469	(0.0003)*
2_D - 3_D	-3.674234	(0.0125)*
3_13C - 3_D	3.674234	(0.0063)*

Day 38:

Pairwise comparisons	t statistic	(Holm adjusted p-value)
2_13C - 2_D	11.02270	(4.1e-06)*
2_13C - 3_13C	3.674234	(0.0188)*
2_D - 3_13C	-7.348469	(0.0004)*
2_13C - 3_D	7.348469	(0.0003)*
2_D - 3_D	-3.674234	(0.0125)*
3_13C - 3_D	3.674234	(0.0063)*

4.1.3 Biotic tAAs

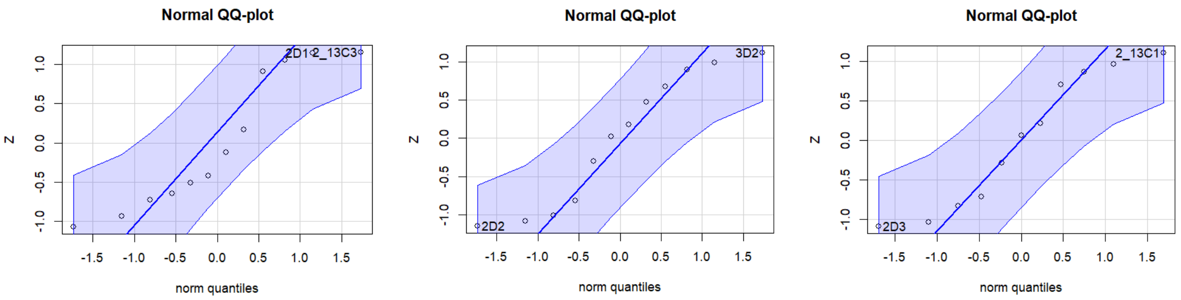

Supplementary Fig. 4: QQ-plots of normalized z-residuals for tAAs in the GLP treatment in biologically active soil fitted with one-way ANOVA. Open dots show the normalized residuals of the fitted anova model for day 4 (left), day 18 (middle) and day 38 (right). Blue lines and the blue shaded areas indicate the reference for a normal distribution with 95% confidence interval. The two data points with the largest deviation from a theoretical normal distribution are labeled. norm quantiles: quantiles normalized to the standard normal distribution (mean = 0, standard deviation = 1), z: z-residuals standardized to the standard normal distribution.

```
> kruskal.test (`day 4` ~ label, data = biotic_tAAs_GLP)
```

Kruskal-Wallis rank sum test

data: day 4 by label

Kruskal-Wallis chi-squared = 10.385, df = 3, p-value = 0.01556

data: day 18 by label

Kruskal-Wallis chi-squared = 9.4615, df = 3, p-value = 0.02374

data: day 38 by label

Kruskal-Wallis chi-squared = 8.197, df = 3, p-value = 0.04211

Post-hoc comparison:

```
conover.test(biotic_tAAs_GLP$day18, biotic_tAAs_GLP$Label, method="holm", list=TRUE, altp=TRUE)
```

day 4

Pairwise comparisons	t statistic	(Holm adjusted p-value)
2_13C - 2D	3.674234	(0.0188)*
2_13C - 3_13C	7.348469	(0.0004)*
2D - 3_13C	3.674234	(0.0125)*
2_13C - 3D	11.02270	(0.0000)*
2D - 3D	7.348469	(0.0003)*
3_13C - 3D	3.674234	(0.0063)*

day 18

Pairwise comparisons	t statistic	(Holm adjusted p-value)
2_13C - 2D	3.872983	(0.0236)*
2_13C - 3_13C	3.098386	(0.0441)*
2D - 3_13C	-0.774596	(0.4609)
2_13C - 3D	6.971370	(0.0007)*
2D - 3D	3.098386	(0.0294)*
3_13C - 3D	3.872983	(0.0189)*

day 38

Pairwise comparisons	t statistic	(Holm adjusted p-value)
2_13C - 2D	2.425356	(0.1372)
2_13C - 3_13C	4.608176	(0.0123)*
2D - 3_13C	2.182820	(0.1307)
2_13C - 3D	4.880935	(0.0108)*
2D - 3D	2.711630	(0.1205)
3_13C - 3D	0.759256	(0.4725)

4.1.4 Biotic glycine

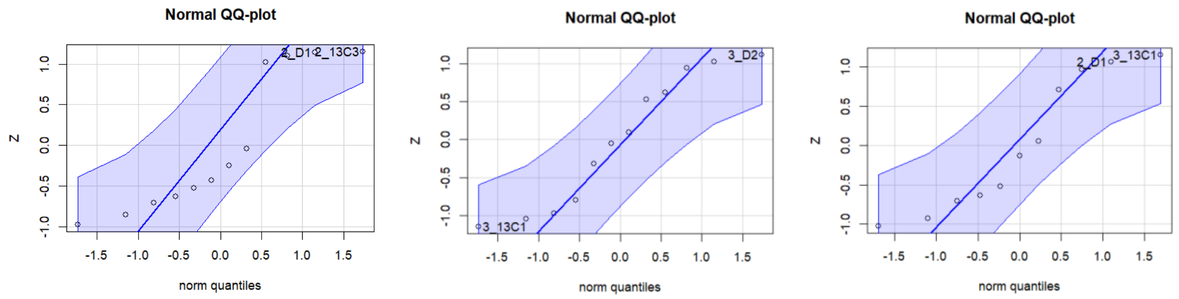

Supplementary Fig. 5: QQ-plots of normalized z-residuals for glycine in the GLP treatment in biologically active soil fitted with one-way ANOVA. Open dots show the normalized residuals of the fitted anova model for day 4 (left), day 18 (middle) and day 38 (right). Blue lines and the blue shaded areas indicate the reference for a normal distribution with 95% confidence interval. The two data points with the largest deviation from a theoretical normal distribution are labeled. norm quantiles: quantiles normalized to the standard normal distribution (mean = 0, standard deviation = 1), z: z-residuals standardized to the standard normal distribution.

Kruskal-Wallis rank sum test

data: day 4 by label
Kruskal-Wallis chi-squared = 9.4615, df = 3, p-value = 0.02374
data: day 18 by label
Kruskal-Wallis chi-squared = 9.4615, df = 3, p-value = 0.02374
data: day 38 by label
Kruskal-Wallis chi-squared = 7.7121, df = 3, p-value = 0.05235

Post-hoc comparison:

```
conover.test(GLP_glycine$`day 4`, GLP_glycine$`Label, method="holm", list=TRUE, altp=TRUE)
```

day 4:

Pairwise comparisons	t statistic	(Holm adjusted p-value)
2_13C - 2_D	-0.774596	(0.4609)
2_13C - 3_13C	3.098386	(0.0441)*
2_D - 3_13C	3.872983	(0.0189)*
2_13C - 3_D	5.422176	(0.0031)*
2_D - 3_D	6.196773	(0.0016)*
3_13C - 3_D	2.323790	(0.0973)

day 18:

Pairwise comparisons	t statistic	(Holm adjusted p-value)
2_13C - 2_D	2.323790	(0.0973)
2_13C - 3_13C	5.422176	(0.0031)*
2_D - 3_13C	3.098386	(0.0441)*
2_13C - 3_D	6.196773	(0.0016)*
2_D - 3_D	3.872983	(0.0189)*
3_13C - 3_D	0.774596	(0.4609)

4.2 2,4-D

4.2.1 Abiotic NERs

```
> t.test (day 0 ~ label, data = abiotic_NERs_2,4-D, alternative = "two.sided", paired = FALSE, var.equal = F
ALSE)
```

Welch Two Sample t-test
Data: day 0 by label
t = 0.36517, df = 2.5935, **p-value = 0.7427**
Data: day 18 by label
t = -1.243, df = 2.3722, **p-value = 0.3226**
Data: day 36 by label
t = -2.0226, df = 3.5819, **p-value = 0.1213**

4.2.2 Biotic NERs

```
> t.test (day 16 ~ label, data = `2,4-D_NERs`, alternative = "two.sided", paired = FALSE, var.equal =
FALSE)
```

Welch Two Sample t-test
Data: day 16 by label
t = 15.368, df = 3.9382, **p-value = 0.000116**
Data: day 36 by label
t = 9.7412, df = 3.4206, **p-value = 0.0013**

4.2.3 Biotic tAAs

```
> t.test (day 16 ~ label, data = `2,4-D_tAAs`, alternative = "two.sided", paired = FALSE, var.equal =
FALSE)
```

Welch Two Sample t-test
Data: day 16 by label
t = 15.714, df = 2.0809, **p-value = 0.003398**
Data: day 36 by label
t = 15.634, df = 2.157, **p-value = 0.002934**

4.3 SMX

4.3.1 Abiotic NERs

```
> t.test (day 0 ~ label, data = abiotic_NERs_SMX, alternative = "two.sided", paired = FALSE, var.equal =
FALSE)
```

Welch Two Sample t-test
Data: day 0 by label
t = 3.4193, df = 2.8253, **p-value = 0.04585**
Data: day 36 by label
t = 7.645, df = 2.7095, **p-value = 0.006578**
Data: day 72 by label
t = 0.86647, df = 2.0796, **p-value = 0.4745**

4.3.2 Biotic NERs

> t.test (day 0 ~ label, data = biotic_NERs_SMX, alternative = "two.sided", paired = FALSE, var.equal = FALSE)

Welch Two Sample t-test

Data: day 0 by label

t = 3.301, df = 2.2987, **p-value = 0.06678**

Data: day18 by label

t = 7.3699, df = 2.8858, **p-value = 0.005886**

Data: day 36 by label

t = 3.4531, df = 2.6067, **p-value = 0.05062**

Data: day 72 by label

t = 5.226, df = 3.2077, **p-value = 0.01151**

4.4 P-value adjustment (Welch-tests)

Supplementary Table 3: P-values of Welch tests and adjusted values (p_{adj}) after Holm-Bonferroni correction for multiple testing for 2,4-D and SMX.

Comparison D vs ^{13}C	p (Welch test)	p_{adj} (Holm)	Significance
Abiotic_NERs_2,4-D_day 0	0.7427	0.968	ns
Abiotic_NERs_2,4-D_day 18	0.3226	0.968	ns
Abiotic_NERs_2,4-D_day 36	0.1213	0.485	ns
Abiotic_NERs_SMX_day 0	0.0459	0.321	ns
Abiotic_NERs_SMX_day 36	0.0066	0.059	ns
Abiotic_NERs_SMX_day 72	0.4745	0.968	ns
Biotic_NERs_2,4-D_day 16	0.0001	0.002	**
Biotic_NERs_2,4-D_day 36	0.0013	0.017	*
Biotic_NERs_SMX_day 0	0.0668	0.334	ns
Biotic_NERs_SMX_day 18	0.0059	0.059	ns
Biotic_NERs_SMX_day 36	0.0506	0.321	ns
Biotic_NERs_SMX_day 72	0.0115	0.092	ns
Biotic_tAAs_2,4-D_day 16	0.0034	0.037	*
Biotic_tAAs_2,4-D_day 36	0.0029	0.035	*

* p<0.05, **p<0.01, *** p<0.001, ns: not significant

Supplementary Note 5: Calculation of $^{13}C_{biogenic\ NERs}$ using the microbial turnover to biomass (MTB) model

The MTB model estimates microbial biomass growth on a biodegradable substrate based on empirical and thermodynamic considerations of the energy and carbon demands for biomass synthesis [1,17]. It can thus calculate an upper and lower bound for bioNER formation from the theoretical growth yield of the substrate and measured $^{13}CO_2$ evolution [1,18] (**Supplementary Table 4**).

Supplementary Table 4: **Cumulative endpoint mineralization of $^{13}C_{6\text{-}2,4\text{-}D}$ (day 36), 2-$^{13}C_{GLP}$ (day 38), 3-$^{13}C_{GLP}$ (day 38) and $^{13}C_{6\text{-}SMX}$ (day 72) used for the MTB calculations.**

Substrate	Cumulative mineralization ($^{13}CO_2$)
$^{13}C_{6\text{-}2,4\text{-}D}$	78 ± 8.8
2-$^{13}C_{GLP}$	40 ± 12
3-$^{13}C_{GLP}$	50 ± 17
$^{13}C_{6\text{-}SMX}$	2.3 ± 0.5

The MTB approach was found to be a robust model for predicting bioNER formation in terms of validity, accuracy and precision, with a mean absolute error of 5% of applied label (range 0.3 to 16%) between predicted and measured bioNER across 16 diverse chemicals [18]. Hence, feasibility of the experimental results from this study were compared to predicted bioNER formation according to the MTB approach.

The following redox reactions were assumed to describe the substrate turnover of 2,4-D (**Supplementary Equation 10**) [1] and SMX (**Supplementary Equation 11**) [19]:

2,4-D: $C_8H_6O_3Cl_2 + 7.5O_2\ (g) \leftrightharpoons 8CO_2\ (g) + 2H_2O\ (l) + 2HCl$ **(10)**

SMX: $C_{10}H_{11}N_3O_3S + 10.5O_2\ (g) \leftrightharpoons 10CO_2\ (g) + 3NH_3\ (l) + H_2SO_4\ (l)$ **(11)**

GLP can be either degraded via the AMPA pathway (**Supplementary Equation 12**), or the sarcosine pathway (**Supplementary Equation 13**), leading to the formation of glycine[2,20]. In this study, both pathways played a role as demonstrated by separate isotope-labeling of GLP at the second or third carbon atom. The AMPA pathway was evidenced by the formation of $^{13}C_{AMPA}$ from 3-C_{GLP}, while the sarcosine pathway was indirectly identified from the difference in $^{13}C_{glycine}$ formation between 2-C_{GLP} and 3-C_{GLP}.Therefore, for GLP two separate estimates of bioNER formation were calculated based on the two pathways. Additionally, turnover of glycine was calculated. It should, however, be noted that Brock et al. [17] found higher discrepancies between predicted and experimental growth yield for this biomolecule.

GLP (AMPA pathway): $C_3H_8NO_5P + 2H_2O\ (l) \leftrightharpoons CH_6NO_3P + 2CO_2\ (g) + 6H^+ + 6e^-$ **(12)**

GLP (sarcosine pathway):

$C_3H_8NO_5P + 5H_2O\ (l) \leftrightharpoons 3CO_2\ (g) + NH_4^+(l) + H_3PO_4\ (l) + 11H^+ + 12e^-$ **(13)**

Glycine: $C_2H_5NO_2 + 2H_2O\ (l) \leftrightharpoons 2CO_2\ (g) + NH_4^+\ (l) + 5H^+ + 6e^-$ (14)

According to the MTB model [17], the microbial growth yield on a given substrate (Y) in grams of cell carbon per g of substrate carbon is calculated as:

$$Y\left[\frac{g\ C_{cell}}{g\ C_{substrate}}\right] = \left(\frac{\frac{n_{bio}}{n}\frac{\Delta G_r^{0\prime}}{\Delta G_{ATP}} \times Y_{ATP}}{\frac{M_C}{f_C} \times n_C + \frac{n_{bio}}{n}\frac{\Delta G_r^{0\prime}}{\Delta G_{ATP}} \times Y_{ATP}}\right) \quad (15)$$

Here $\mathbf{n_{bio}}$ is the number of bioavailable electrons (assumed to be twice the number of C-H bonds in the substrate) and **n** the total number of electrons in the redox reaction, which is calculated from the oxidation state of the carbon atoms in the substrate ($OS_{substrate}$) during its complete mineralization to CO_2 as n = n_C * (4- OS substrate). $\mathbf{\Delta G_r^{0\prime}}$ is the Gibbs free energyy of the balanced redox reaction at standard physiological conditions (here referring to pH=7, ionic strength = 0.1 M, and activity of products and educts = 1 mM [2]); $\mathbf{\Delta G_{ATP}}$ the microbial energy demand for the conversion of one mol of ADP to ATP (assumed to be 80 kJ mol^{-1} in microbial cells[1]), $\mathbf{M_C}$ the molar mass of carbon [12.01 g mol^{-1}], $\mathbf{f_C}$ the fraction of carbon per microbial cell dw [~0.53 g C (g cell dw)$^{-1}$] [1]; and $\mathbf{n_C}$ the number of carbon atoms in the substrate.

The Gibbs free energy associated with the redox reaction is calculated as:

$$\Delta G_r^{0\prime} = \Delta G_{products}^0 - \Delta G_{educts}^0 \quad (16)$$

Values for the Gibbs energy of the involved substrates and turnover products are given in **Supplementary Table 5**. Accordingly, the Gibbs energy of the redox reactions was derived for 2,4-D and SMX. Values for GLP and glycine were directly taken from Brock et al. [2]

$\Delta G_r^{0\prime}{}_{2,4D}$ = [8(-394.4)+2(-237.2)+2(-131.23)] - [-248.8 +7.5(0)] = -3643.26 kJ mol^{-1}

$\Delta G_r^{0\prime}{}_{SXM}$ =[10(-394.4)+3(-26.5)+(-744.53)] – [113.43+10.5(0)] = -4881.46 kJ mol^{-1}

$\Delta G_r^{0\prime}{}_{GLP,\ sarcosine}$ = -536.2 kJ mol^{-1} [(2)]

$\Delta G_r^{0\prime}{}_{GLP,\ AMPA}$ = -203.4 kJ mol^{-1} [(2)]

$\Delta G_r^{0\prime}{}_{Glycine}$ = -235.7 kJ mol^{-1} [(2)]

Supplementary Table 5: **Values of Gibbs energy of formation.** Unless otherwise specified, they refer to standard metabolic conditions (pH = 7, I = 0.1 M, activity of products and educts = 1 mM [except H^+]). Values for 2,4-D and glycine were calculated using eQuilibrator (http://equilibrator.weizmann.ac.il).

**Molecule**	**$\Delta G_r0'$**	**Reference**
**2,4-D**	-248.8	eQuilibrator
**GLP**	-896.6	[2]
**Glycine**	-173.9	eQuilibrator
**SMX**	113.43*	[19,21]
**O_2**	0	[1]
**H^+ (pH 7)**	-39.87	[1]
**CO_2**	394.4	[1]
**H_2O**	237.2	[1]
**HCl**	-131.23	[1]
**NH_3**	-26.5	[1]
**H_3PO_4**	-1111.9	[22]

* conditions not specified, but MTB was found to be robust with regard to changes in the substrate Gibbs energy from standard conditions [17]

Once Y is determined (for relevant input parameters see **Supplementary Table 6**), the maximum and minimum amounts of bioNER formation can be estimated. These correspond to either to a complete catabolic substrate conversion to CO_2, typically found in short-term experiments ($bioNER_{max}$, **Supplementary Equation 17**), or to partly anabolic and partly catabolic conditions ($bioNER_{min}$, **Supplementary Equation 18**). Typically, 50% of decaying microbial biomass is assumed to be mineralized to CO_2, yielding a fraction (**f**) of 50% of the decaying microbial biomass retained in bioNERs either in form of living biomass or stabilized in soil organic matter [1,18,19].

$$bioNER_{max} = \frac{Y}{1-Y} \times [CO_2] \qquad (17)$$

$$bioNER_{min} = \frac{f \times Y}{(1-Y)+(1-f)\times Y} \times [CO_2] \qquad (18)$$

Supplementary Table 6: Input parameters and results of MTB modelling.

Parameter	Substrate						
	$^{13}C_{6\text{-}SMX}$	$^{13}C_{6\text{-}2,4\text{-}D}$	2-$^{13}C_{GLP}$ 1)	2-$^{13}C_{GLP}$ 2)	3-$^{13}C_{GLP}$ 1)	3-$^{13}C_{GLP}$ 2)	Glycine
**M_S [g mol^{-1}]**	260.08	224.98	169.01	169.01	169.01	169.01	76.03
**n_{bio}**	16	10	8	8	8	8	4
**n_C**	10	8	3	3	3	3	2
**n**	34	30	10	10	10	10	6
**$\Delta G_r^{0'}$ [kJ mol^{-1}]**	-4881.5	-3643.3	-536.2	-203.4	-536.2	-203.4	-235.7
**Y_{ATP} [g cell dw (mol ATP)$^{-1}$]**	5	5	5	5	5	5	5
**Y [g C_{cell} (g $C_{substrate}$)$^{-1}$]**	0.39	0.30	0.28	0.13	0.28	0.13	0.18
**CO_2 [% applied ^{13}C]**	2.33	77.82	40.44	40.44	49.53	49.53	40.44
**$bioNER_{max}$ [% applied ^{13}C]**	**1.48**	**32.58**	**15.95**	**6.05**	**19.53**	**7.41**	**8.76**
**$bioNER_{min}$ [% applied ^{13}C]**	**0.56**	**13.47**	**6.66**	**2.81**	**8.16**	**3.45**	**3.95**

M_s: molar mass of substrate; **n_{bio}** the number of bioavailable electrons; **n:** total number of electrons in the redox reaction, **n_C**: number of carbon atoms in the substrate; **$\Delta G_r^{0'}$**: Gibbs free energy of the balanced redox reaction describing the substrate breakdown; **Y_{ATP}**: bacterial growth yield from ATP, assumed to be 5 g cell dw (mol ATP)$^{-1}$ due to the non-'sugar-like' structure of SMX, 2,4-D, GLP and glycine [1,17]; **Y**: bacterial growth yield on a given substrate utilized as both an electron donor and a carbon source[1]; **CO_2**: substrate-derived $^{13}CO_2$ evolution at the end of the incubation experiment [% of applied g of ^{13}C]; **$bioNER_{max}$** = maximum estimate of bioNER formation (exlcusively catabolic carbon utilization); **$bioNER_{min}$**= minimum estimate of bioNER formation. **1)** degradation via the sarcosine pathway, **2)** degradation via the AMPA pathway.

Supplementary Note 6: Assessment of D-label stability in water

Background. Throughout the incubations and mass balance analyses for biodegradability tests stability of the isotope label is crucial. Deuterium (D) labels of the model compounds were therefore attached to C atoms as D bound to O, N or S was shown to exchange more easily with H in water [23,24]. In contrast, covalent C–H bonds of different organic substrates (glucose, C–$D_{2\text{-alanine}}$ and C–$D_{2\text{-glycine}}$) were shown to remain stable in soil incubations and even under harsh treatments of acid hydrolysis and esterification. D/H exchange was only observed during enzymatic cleavage by microorganisms, suggesting high stability of C–D bonds [23,25]. Nevertheless, it is important to confirm the stability of the C–D bonds of $D_{3\text{-}2,4\text{-}D,}$ 2-C–$D_{2\text{-}GLP}$, 3-C–$D_{2\text{-}GLP}$ and $D_{4\text{-}SMX}$ as very slow D/H exchange can also occur in aliphatic and aromatic molecules [26].

Approach. To assess the stability of the C–D bonds of the model substances against H exchange in water, triplicate sets of aqueous solutions of each unlabeled, ^{13}C- and D-labeled chemicals (100 µg L^{-1} for 2,4-D and SMX, and 100 mg L^{-1} for GLP) were incubated in the dark at 20°C for 6 days. The relative abundances of their isotopologues containing different amounts of D (e.g. unlabeled 2,4-D, $D_{1\text{-}2,4\text{-}D}$, $D_{2\text{-}2,4\text{-}D}$ and $D_{3\text{-}2,4\text{-}D}$, etc.) or ^{13}C were quantified by liquid chromatography-high resolution mass spectrometry (LC-HRMS) after 0 and 6 days to check for potential loss of the D-label. In these tests, the unlabeled and ^{13}C-labeled compounds served as controls. All compounds were analyzed with a Thermo Ultimate 3000 LC coupled to a Thermo QExactive Plus high-resolution mass spectrometer. For analysis of GLP and SMX, a Thermo Acclaim Trinity Q1 column (100 × 3 mm, 3 µm particle size) was used with a gradient separation employing 25 mM NH_4-formate buffer (pH 3.5) and acetonitrile. The MS was operated in positive electrospray ionization (ESI) mode for SMX and in negative mode for GLP. 2,4-D was analyzed using a gradient separation with water/methanol (both containing 0.1% v/v formic acid) on a Phenomemex Kinetex C18 EVO column (50 × 2.1 mm, 2.6 µm particle size). The MS was operated in negative ESI mode. All compounds were analyzed in full scan experiments at a nominal resolving power of 70,000 (referencing to m/z 200). The results from the stability tests are shown as mean peak areas of triplicate samples on day 0 (without incubation) and on day 6 in **Supplementary Table 7**.

2,4-D. The initial standard solution of $D_{3\text{-}2,4\text{-}D}$ on day 0 contained additional ions corresponding to $D_{4\text{-}2,4\text{-}D}$ (m/z 222.9871), $D_{2\text{-}2,4\text{-}D}$ (m/z 220.9746), and $D_{1\text{-}2,4\text{-}D}$ (m/z 219.9683) as well as noise signals for unlabeled 2,4-D and $^{13}C_{6\text{-}2,4\text{-}D}$. The noise signals were also observed in blank injections (data not shown) and were therefore not further considered. Direct quantification of the impurities was not possible due to a lack of reference standards; however, their responses were assumed to be similar to that of the native $D_{3\text{-}2,4\text{-}D}$. Impurities made up roughly 6% of all 2,4-D isotopologues in the $D_{3\text{-}2,4\text{-}D}$

standard solution (**Supplementary Table 7**). $D_{4\text{-}2,4\text{-}D}$ contributed roughly 1.1%, $D_{2\text{-}2,4\text{-}D}$ 4.3% and $D_{1\text{-}2,4\text{-}D}$ 0.05%. These findings somewhat contradict the manufacturer purity specification of >99%, however, it should be noted that the compound had been stored for over one year after initial opening, and possibly some degradation had occurred. Moreover, low-level D/H exchange may have occurred in the ion source. Slightly higher mean areas of $D_{4\text{-}2,4\text{-}D}$, $D_{2\text{-}2,4\text{-}D}$ as well as the native $D_{3\text{-}2,4\text{-}D}$ were observed after 6-day incubation. For $D_{2\text{-}2,4\text{-}D}$ the mean area increased by about 2% from 5.72E+06 to 5.84E+06 (3% RSD in both cases) while the area of $D_{4\text{-}2,4\text{-}D}$ was almost unchanged (about 0.4% increase). In both cases, however, the variation of the measurement was larger than the observed change, so these differences are not deemed significant. Moreover, a roughly 2% increase was also observed for the mean area of the $D_{3\text{-}2,4\text{-}D}$, which increased from 1.27E+08 to 1.30E+08 between day 0 and day 6. Theses slight increases may have been caused by gradual water evaporation from the incubated solution, which would have slightly concentrated the dissolved compounds. A similar effect was also observed for the incubated $^{13}C_{6\text{-}2,4\text{-}D}$ solution, where both the $^{13}C_{6\text{-}2,4\text{-}D}$ and its $^{13}C_{5}$-impurity had increased by around 4-5% after 6 days. In the unlabeled 2,4-D solution, the abundance of the natural $^{13}C_{1}$-isotopologue also increased between day 0 and day 6 while no increase in the average area of 2,4-D was observed (-0.3% change between day 0 and day 6; **Supplementary Table 7**). However, the relative standard deviations (RSDs) were rather large with 13-15% which may have masked the effect. The $D_{3\text{-}2,4\text{-}D}$ standard, therefore, appeared completely stable in the aqueous solution over 6 days and no significant D exchange should have occurred throughout the 36-day soil incubation experiment.

GLP. The solutions of both 2-C–$D_{2\text{-}GLP}$, as well as 3-C–$D_{2\text{-}GLP}$, contained impurities from unlabeled GLP, $D_{1\text{-}GLP}$ and $D_{3\text{-}GLP}$. The 2-C–$D_{2\text{-}GLP}$ solution contained around 93% of the native compound, around 6% of 2-C–$D_{1\text{-}GLP}$ and 0.2% and 0.03% of unlabeled and 2-C–$D_{3\text{-}GLP}$, respectively (**Supplementary Table 7**). Like for 2,4-D, this was below the manufacturer purity specification suggesting that the compound may have slowly degraded under long-term storage conditions. The mean areas of unlabeled, 2-C–$D_{1\text{-}}$, and 2-C–$D_{2\text{-}GLP}$ all decreased after 6 days by 23%, 9% and 13%, respectively, while only the mean area of 2-C–$D_{3\text{-}GLP}$ increased slightly. However, as the variation of peak areas, especially on day 0, was in a similar range (between 5-12% RSD for both 2-C–$D_{2\text{-}GLP}$ and 2-C–$D_{1\text{-}GLP}$ and 6-18% for unlabeled and 2-C–$D_{3\text{-}GLP}$), the observed changes in mean areas do not appear to be significant. Regarding the overall composition of the solution, the relative abundance of 2-C–$D_{2\text{-}GLP}$ decreased very slightly from 93.3 to 93.0% over 6 days while that of 2-C–$D_{1\text{-}GLP}$ increased slightly from 6.4% to 6.7%. Scaled to the length of the soil incubation experiment, this would result in less than 2% 2-C–$D_{2\text{-}GLP}$ degradation or 2-C–$D_{1\text{-}GLP}$ formation. A similar degradation of roughly 3% was calculated for both 2-$^{13}C_{GLP}$ and 3-$^{13}C_{GLP}$ which should be inherently more stable, while

almost no change in composition was observed for the unlabeled GLP solution (**Supplementary Table 7**).

The purity of the 3-C–$D_{2\text{-GLP}}$ solution was over 99.5% on both day 0 and 6, with only minor impurities of $D_{3\text{-GLP}}$, $D_{1\text{-GLP}}$ and GLP (**Supplementary Table 7**). A slight increase of the mean 3-C–$D_{2\text{-GLP}}$ area was observed after 6 days (+4%) while 3-C–$D_{1\text{-GLP}}$ decreased by 6.7%. This equates to about 0.2% increase in the relative abundance of 3-C–$D_{2\text{-GLP}}$ in the soil incubation but is not considered significant. Overall, abiotic D/H exchange, therefore, does not seem to have affected the results of 2-C–$D_{2\text{-GLP}}$ and 3-C–$D_{2\text{-GLP}}$ turnover in the soil incubation experiments.

SMX. The $D_{4\text{-SMX}}$ stock solution was almost completely neat on day 0; only one of the three replicates contained around 0.21% of $D_{3\text{-SMX}}$. After the 6-day incubation, there was a strong increase in $D_{3\text{-SMX}}$. Treating missing peaks as zero, the relative abundance of the $D_{3\text{-SMX}}$ impurity increased from on average about 0.07% on day 0 to roughly 0.25% after six days (**Supplementary Table 7**). Although this suggests instability of the $D_{4\text{-SMX}}$, the results are not fully conclusive. Firstly, non-detectable $D_{3\text{-SMX}}$ peaks in two replicates of day zero may be due to the instrumental detection limit rather than the complete absence of the compound, so that the mean area value of $D_{3\text{-SMX}}$ on day 0 is rather uncertain. Notably, the increase in the impurity content was very small (0.02%) in the replicate that already contained visible amounts of $D_{3\text{-SMX}}$ on day 0. Moreover, also the average area of $D_{4\text{-SMX}}$ increased by about 5% from 7.11E+07 on day 0 to 7.45E+07 after 6 days (4-6% RSD). Increases in the abundance of both the native compound as well as present impurities were also observed for unlabeled SMX and $^{13}C_{6\text{-SMX}}$. In both cases, the average area of the native compound increased by around 9% and that of the different impurities by 5-15% (**Supplementary Table 7**). Similar to the 2,4-D solutions, this increase may have been caused by the slow evaporation of water over the incubation period. Nevertheless, because the increase in $D_{3\text{-SMX}}$ appeared to be rather large, we cannot exclude that one of the deuterium atoms of $D_{4\text{-SMX}}$ was exchanged in the aqueous solution. As Kostyukevic et al. noted, electron donor groups in the aromatic ring (such as the –NH_2 group in SMX) can facilitate D/H exchange [26]. However, based on our measurements, the potential D exchange reaction would have taken place rather slowly: after 6 days, less than roughly 0.2% additional $D_{3\text{-SMX}}$ impurity was formed. Assuming a constant rate of the D/H exchange reaction, this would amount to no more than roughly 2% of $D_{4\text{-SMX}}$ degradation throughout the 72-day soil incubation experiment. Although the compound should ideally be completely stable, its potential degradation in aqueous solution was so slow that it seems unlikely to have affected the results of this study.

Conclusion. After 6-day incubation of aqueous stock solutions of the deuterated test compounds at 20°C, no significant release of D from either the second or third aliphatic C atom of $D_{2\text{-GLP}}$ or the aromatic ring of $D_{3\text{-2,4-D}}$ was observed. $D_{4\text{-SMX}}$ seemed to degrade at a very slow rate. However, for

all three deuterated model compounds, the expected degradation throughout the 36- to 72-day soil incubation would remain below 3%. Abiotic degradation due to D/H exchange therefore seems to have played no significant role in the compound turnover in the model soil for the duration of the incubation experiments of this study.

Supplementary Table 7: Stability assessment of the test compounds in aqueous solution after 6-day incubation at 20°C. The abundance of the standards (highlighted) and their respective isotopic impurities are given as mean peak areas and relative standard deviation (RSD) of three independent replicates. The % change indicates the relative change in the mean area over 6 days in an aqueous solution. Standard purity and the abundance of impurities after 0 and 6 days were estimated assuming identical signal intensity from all isotopologues of one compound. Assuming linear compound degradation kinetics (e.g. due to D/H exchange), the difference in the standard purity over 6 days was scaled to the respective length of the incubation in the soil study to estimate the extent of potential abiotic compound degradation in soil.

Incubated standard solution	Detected ions		Peak areas					Approximate % standard (im)purity		
	m/z	Compound	Day 0 mean	Day 0 RSD%	Day 6 mean	Day 6 RSD%	% change	Day 0	Day 6	Estimated change in soil study (Δ%)
**100 µg/L**	**[M-H]**									**36 days**
**2,4-D**	**218.962**	**2,4-D**	**2.45E+10**	**15**	**2.44E+10**	**13**	**-0.3**	**91.86**	**89.27**	**-15.5**
	219.96535	$^{13}C_{1\text{-}2,4\text{-}D}$	2.10E+09	12	2.87E+09	1	37.0	7.86	10.49	15.8
	220.9687	$^{13}C_{2\text{-}2,4\text{-}D}$	7.66E+07	16	6.70E+07	78	-12.5	0.29	0.24	-0.3
**$^{13}C_{6\text{-}2,4\text{-}D}$**	222.9754	$^{13}C_{4\text{-}2,4\text{-}D}$	5.63E+04	70	3.38E+04	52	-39.9	0.04	0.03	-0.1
	223.9788	$^{13}C_{5\text{-}2,4\text{-}D}$	4.96E+06	3	5.21E+06	3	4.9	3.83	3.88	0.3
	**224.9821**	**$^{13}C_{6\text{-}2,4\text{-}D}$**	**1.25E+08**	**1**	**1.29E+08**	**2**	**3.6**	**96.13**	**96.1**	**-0.2**
**$D_{3\text{-}2,4\text{-}D}$**	219.9683	$D_{1\text{-}2,4\text{-}D}$	6.81E+04	45	6.64E+04	41	-2.5	0.05	0.05	0.0
	220.9746	$D_{2\text{-}2,4\text{-}D}$	5.72E+06	3	5.84E+06	3	2.1	4.25	4.26	0.1
	**221.9808**	**$D_{3\text{-}2,4\text{-}D}$**	**1.27E+08**	**1**	**1.30E+08**	**0**	**1.9**	**94.56**	**94.57**	**0.1**
	222.9871	$D_{4\text{-}2,4\text{-}D}$	1.54E+06	3	1.54E+06	10	0.4	1.14	1.13	-0.1
**100 mg/L**	**[M-H]**									**38 days**
**GLP**	**168.0067**	**GLP**	**6.24E+09**	**15**	**8.39E+09**	**8**	**34.4**	**96.78**	**96.81**	**0.1**
	169.0101	$^{13}C_{1\text{-}GLP}$	2.07E+08	16	2.77E+08	8	33.5	3.21	3.19	-0.1
	170.0136	$^{13}C_{2\text{-}GLP}$	3.78E+05	11	3.54E+05	14	-6.5	0.006	0.004	-0.01
**2-$^{13}C_{1\text{-}GLP}$**	168.0067	GLP	2.81E+07	14	2.89E+07	15	2.7	0.68	0.66	-0.1
	**169.0101**	**$^{13}C_{1\text{-}GLP}$**	**4.02E+09**	**12**	**4.24E+09**	**3**	**5.5**	**97.89**	**97.38**	**-3.2**
	170.0136	$^{13}C_{2\text{-}GLP}$	5.85E+07	12	8.51E+07	3	45.6	1.42	1.96	3.4
**3-$^{13}C_{1\text{-}GLP}$**	168.0067	GLP	3.38E+07	7	3.42E+07	8	1	0.51	0.5	-0.1
	**169.0101**	**$^{13}C_{1\text{-}GLP}$**	**6.54E+09**	**5**	**6.61E+09**	**8**	**1.1**	**97.81**	**97.35**	**-2.9**
	170.0136	$^{13}C_{2\text{-}GLP}$	1.12E+08	30	1.46E+08	8	29.5	1.68	2.14	2.9

Incubated standard solution	Detected ions		Peak areas					Approximate % standard (im)purity		
			Day 0		Day 6		%			Estimated change in
	m/z	Compound	mean	RSD%	mean	RSD%	change	Day 0	Day 6	soil study (Δ%)
**2-C–$D_{2\text{-GLP}}$**	168.0067	GLP	2.05E+07	13	1.56E+07	6	-23.5	0.2	0.17	-0.2
	169.013	$D_{1\text{-GLP}}$	6.67E+08	9	6.07E+08	5	-9	6.44	6.75	2.0
	**170.0193**	**$D_{2\text{-GLP}}$**	**9.66E+09**	**12**	**8.37E+09**	**5**	**-13.4**	**93.33**	**93.04**	**-1.8**
	171.0256	$D_{3\text{-GLP}}$	2.75E+06	18	3.31E+06	7	20.3	0.03	0.04	0.1
**3-C–$D_{2\text{-GLP}}$**	168.0067	GLP	1.12E+06	8	1.42E+06	27	26.3	0.011	0.013	0.01
	169.013	$D_{1\text{-GLP}}$	1.59E+07	18	1.48E+07	10	-6.7	0.15	0.14	-0.1
	**170.0193**	**$D_{2\text{-GLP}}$**	**1.05E+10**	**8**	**1.09E+10**	**8**	**4**	**99.54**	**99.58**	**0.3**
	171.0256	$D_{3\text{-GLP}}$	3.08E+07	11	3.02E+07	10	-2	0.29	0.28	-0.06
**100 µg/L**	**[M+H]**									**72 days**
**SMX**	**254.0594**	**SMX**	**1.43E+08**	**10**	**1.56E+08**	**3**	**9**	**90.77**	**90.34**	**-5.1**
	255.0628	$^{13}C_{1\text{-SMX}}$	1.41E+07	0	1.62E+07	0	14.6	8.97	9.39	2.7
	256.0661	$^{13}C_{2\text{-SMX}}$	4.17E+05	13	4.65E+05	11	11.3	0.265	0.27	0.03
**$^{13}C_{6\text{-SMX}}$**	259.0762	$^{13}C_{5\text{-SMX}}$	1.47E+07	3	1.54E+07	1	4.5	4.41	4.25	-1.0
	**260.0795**	**$^{13}C_{6\text{-SMX}}$**	**3.19E+08**	**3**	**3.46E+08**	**1**	**8.5**	**95.59**	**95.75**	**1.9**
**$D_{4\text{-SMX}}$**	257.0782	$D_{3\text{-SMX}}$	4.85E+04	173	1.83E+05	12	277.7	0.07	0.25	1.1
	**258.0845**	**$D_{4\text{-SMX}}$**	**7.11E+07**	**4**	**7.45E+07**	**7**	**4.7**	**99.93**	**99.75**	**-2.1**

Supplementary Note 7: Respiration of soil spiked with 2,4-D, GLP and SMX

Ideally, during the soil incubation experiments a larger soil batch with (relatively) small aliquots sampled per day would have been used, or alternatively separate bottles with sacrificial sampling. However, due to the high costs of the isotope-labeled compounds and limited availability (only <100 mg upon request for custom-synthesis in some cases), the amount of spiked soil had to be minimized. Therefore, we performed incubations with small soil batches (60 g soil wet weight), from which about 6-18 g subsamples were taken on days 4, 16/18 and 36/38 for GLP and 2,4-D and on days 18, 38 and 72 for SMX. Soil samples were taken carefully from different spots within the soil batch to minimize soil disturbance, which could alter soil conditions and thus affect microbial activity and NER formation. For instance, a thorough soil mixing can allocate labile carbon substrates promoting microbial activity [27]; which is demonstrated in an increased soil respiration. Therefore, we also monitored soil respiration at each sampling date (see **Supplementary Fig. 6**) to assess whether soil sampling changed soil microbial activity. Total CO_2 evolved from the added model compounds (2,4-D, GLP or SMX) or other potentially labile carbon substrate(s) in soil was trapped into NaOH-inserts placed inside the soil batch. The CO_2 was quantified with a total inorganic carbon (TIC) analyzer (Multi N/C 2100S, Jena, Germany).

Typically, soil respiration is highest after setting up the soil batches due to the increased availability of existing carbon substrates as well as the newly introduced ones (in this case 2,4-D, GLP or SMX), which can both promote soil microbial activity. Afterwards, soil respiration should decrease if the soil system is undisturbed due to decreasing availability of carbon substrates to microorganisms over time. Soil respiration for each compound (2,4-D, GLP and SMX) was initially highest and then decreased gradually towards the end of incubation suggesting no notable or only minimal soil disturbance after multiple sampling. Due to a specific set-up of the batch series with D-labeled compounds, which contained only inserts with Na_2SO_4 to trap D-water vapor, we were not able to measure total CO_2. All set-ups were handled with the same care while soil sampling; therefore, we also do not expect any notable changes in the soil respiration in batches with the D-labeled compounds. Small differences in soil respiration between labeled test compounds and their unlabeled counterparts are hard to explain. Nevertheless, in this study, unlabeled set-ups served only as a control for isotopic abundance which was nearly identical over all sampling days; therefore, they at most minimally affect the $^{13}C/D_{NERs}$ and $^{13}C/D_{tAAs}$ results shown in this study.

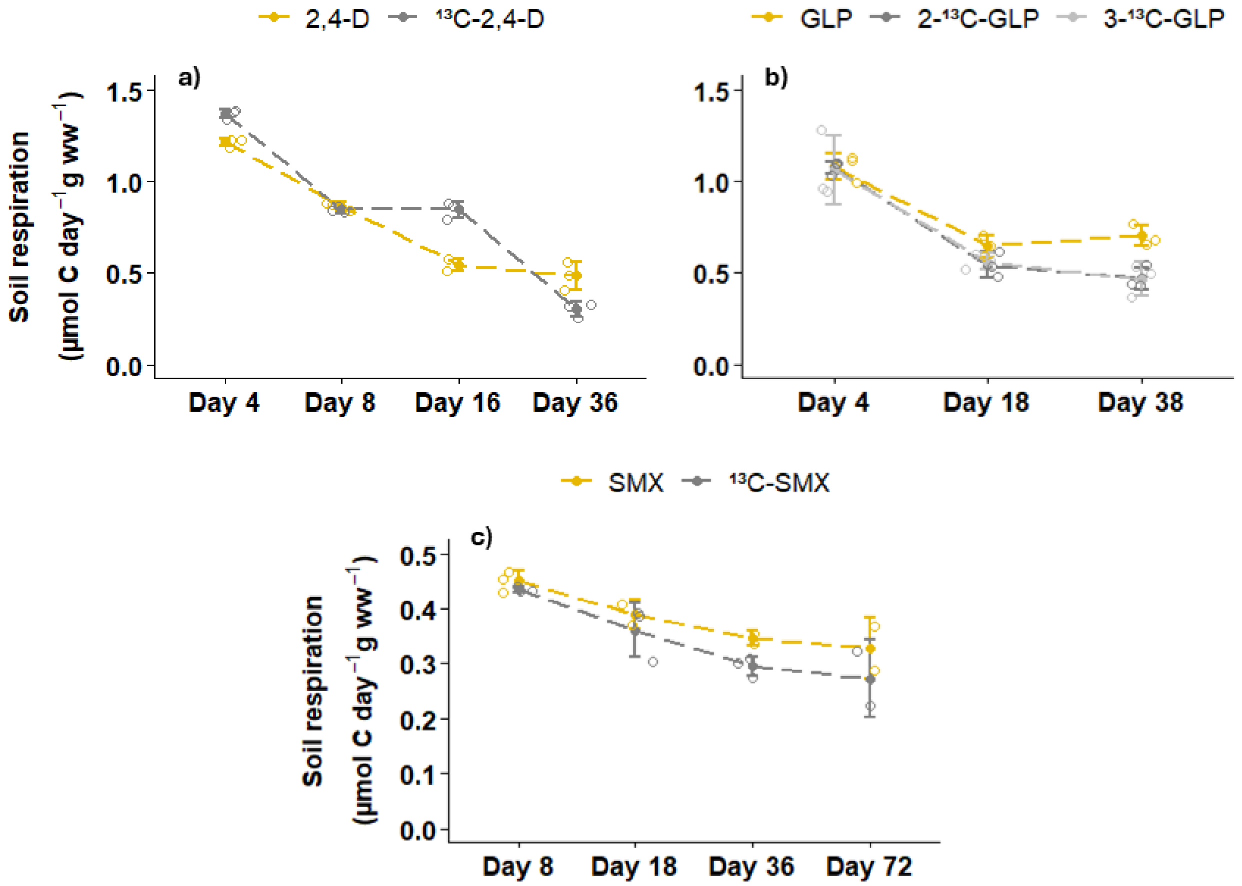

Supplementary Fig. 6: Respiration of soil incubated with (a): 2,4-dichlorophenoxyacetic acid (2,4-D), (b): glyphosate (GLP) and (c): sulfamethoxazole (SMX). Yellow dots: unlabeled model compounds (2,4-D, GLP and SMX), **Grey dots**: ^{13}C-labeled analogue of 2,4-D ($^{13}C_{6\text{-}2,4\text{-}D}$), GLP (two labeling positions: 2-$^{13}C_{GLP}$ and 3-$^{13}C_{GLP}$) and SMX ($^{13}C_{6\text{-}SMX}$). Closed dots and error bars show the mean ± standard deviation and open circles individual replicates. Soil respiration was initially highest for all three tested compounds and it decreased towards the end of incubation (**a**, **b**, **c**).

Supplementary Methods

Supplementary Method 1: Detection limits for total NER and bioNER analysis using ^{13}C and D tracers

For total (bio)NER analysis, two separate limits of detection/quantification (LOD/LOQ) can be derived. Firstly, a LOD/LOQ for the minimum detectable or quantifiable amount (µmol) of total C/H or individual amino acids in dry soil, which coul be calculated from the calibration curve of a standard according to the regression method outlined in DIN32645[28]. However, limits for total C/H or individual AA abundances are not reported here as they were not the main limiting factor during (bio)NER analysis. This is because the total C/H/AA content per sample can be manipulated easily by altering the sample weight for NER analysis by EA-IRMS or the extract and injection volume for AA analysis by GC-(IR)MS. The second LOD/LOQ represent the minimum detectable or quantifiable isotopic enrichment, i.e. the smallest measurable difference between the isotope ratios (at%) in the labeled samples and unlabeled controls. These limits are the most critical for (bio)NER quantification as isotope ratios are determined by soil properties and the amount of applied label but cannot be meaningfully manipulated during analysis. Hence only the LOD/LOQ relevant to the at% readings are reported here. Please note that these do not represent the absolute minimum measurable isotopic abundance in soil but rather the minimum measurable difference between two samples.

We estimated the theoretical instrumental limits of detection (IDL) from the precision of the at% measurements on the IRMS, which is $\leq 7.79\text{x}10^{-5}$ at% D ($\delta D \leq 5‰$) and $\leq 3.28\text{x}10^{-4}$ at% ^{13}C ($\delta^{13}C \leq 0.3‰$)[29]. In line with the procedures laid out by EURACHEM[30], we then applied a generic multiplication factor of 3 to safely distinguish two measurements (labeled and unlabeled) with the given precision. Hence the IDL (in at% enrichment) was derived as three times the instrument standard deviation ($SD_{instrument}$) and was assumed to be equal to the quantification limit as it is already based on the instrument precision. For the test compounds applied in concentrations of 10-50 mg [kg dry soil]$^{-1}$ (equivalent to 0.1-0.5 µmol isotope label [g dry soil]$^{-1}$), this corresponds to a theoretical IDL for NERs ≤5-8% of applied ^{13}C (**Supplementary Table 8a**) and ≤2-5% of applied D (**Supplementary Table 8b**). IDLs for individual AA were calculated in the same way and ranged from ≤0.003-0.18% of applied ^{13}C and ≤0.0004-0.15% of applied D (**Supplementary Table 8c** and **8d**). These estimates are however based on a generic, possibly conservative estimate of the instrument precision and only valid for the specific model soil, test compounds and analytical settings used in this study.

The actual LODs for (bio)NER analysis may differ based on the actual measurement precision and sample inhomogeneity affecting at% readings. We therefore also estimated a more realistic method-

specific detection limit (MDL) based on the standard deviation in the measured at% values in the unlabeled background control over all sampling days as MDL= 3 x $SD_{background}$. The quantification limit is assumed equal to the detection limit, resulting in LOD estimates for NERs equivalent to 3-5% applied D and 4-5% applied ^{13}C for our model compounds, similar to the IDLs. The estimated MDLs for AAs were about one order of magnitude higher than the respective IDLs as they represent variation in biological processes across all sampling time points, which can be much larger than for abiotic processes (**Supplementary Table 8c** and **Supplementary Table 8d**).

Supplementary Table 8a: Estimated detection limits for $^{13}C_{NERs}$

$^{13}C_{NERs}$	2,4-D	GLP	SMX
Spike concentration (mg kg^{-1})	10	50	20
^{13}C /molecule	6	1	6
µmol ^{13}C (g dry soil)$^{-1}$	0.21	0.23	0.36
$SD_{instrument}$ (at% ^{13}C)	≤0.0003	≤0.0003	≤0.0003
IDL (excess at% ^{13}C)	≤0.0018	≤0.0018	≤0.0018
**IDL (% applied ^{13}C)**	**≤6.93**	**≤7.98**	**≤5.09**
$SD_{background}$ (at% ^{13}C)	2.0E-04	1.8E-04	2.7E-04
MDL (excess at% ^{13}C)	6.1E-04	5.5E-04	8.0E-04
**MDL (% applied ^{13}C)**	**4.33**	**4.44**	**4.15**

Supplementary Table 8b: Estimated detection limits for D_{NERs}

D_{NERs}	2,4-D	GLP	SMX
Spike concentration (mg kg^{-1})	10	50	20
D/molecule	3	2	4
µmol D (g dry soil)$^{-1}$	0.1	0.46	0.24
$SD_{instrument}$ (at% D)	≤0.0001	≤0.0001	≤0.0001
IDL (excess at% D)	≤0.0003	≤0.0003	≤0.0003
**IDL (% applied D)**	**≤4.70**	**≤2.21**	**≤4.09**
$SD_{background}$ (at% D)	6.3E-05	9.9E-05	9.6E-05
MDL (excess at% D)	1.9E-04	3.0E-04	2.9E-04
**MDL (% applied D)**	**3.82**	**2.82**	**5.07**

Supplementary Table 8c: Estimated detection limits for $^{13}C_{AAs}$ and D_{AAs} for GLP

$^{13}C_{AA}$	# C atoms	# H atoms	µmol AA/g soil	(% applied ^{13}C)		(% applied D)	
				IDL	MDL	IDL	MDL
**Alanine**	3	7	2.10	**0.027**	**0.145**	**0.008**	**0.056**
**Glycine**	2	5	2.75	**0.024**	**0.233**	**0.007**	**0.060**
**Threonine**	4	9	0.96	**0.017**	**0.739**	**0.004**	**0.035**
**Serine**	3	7	0.94	**0.012**	**0.317**	**0.003**	**0.026**
**Valine**	5	11	1.18	**0.025**	**0.412**	**0.007**	**0.031**
**Leucine**	6	13	1.14	**0.029**	**0.308**	**0.008**	**0.035**
**Isoleucine**	6	13	0.79	**0.020**	**0.328**	**0.005**	**0.043**
**Proline**	5	9	1.23	**0.026**	**0.236**	**0.006**	**0.031**
**Aspartate**	4	7	2.75	**0.047**	**0.719**	**0.010**	**0.078**
**Glutamate**	5	9	2.63	**0.056**	**0.439**	**0.012**	**0.084**
**Phenylalanine**	9	11	0.61	**0.024**	**0.306**	**0.003**	**0.014**
**Tyrosine**	9	11	0.08	**0.003**	**0.068**	**0.000**	**0.046**
**Lysine**	6	14	0.45	**0.012**	**0.138**	**0.003**	**0.019**

Supplementary Table 8d: Estimated detection limits for $^{13}C_{AAs}$ and D_{AAs} for 2,4-D

$^{13}C_{AA}$	# C atoms	# H atoms	µmol AA/g soil	(% applied ^{13}C)		(% applied D)	
				IDL	MDL	IDL	MDL
**Alanine**	3	7	9.64	**0.138**	**0.742**	**0.151**	**1.115**
**Glycine**	2	5	9.49	**0.091**	**0.897**	**0.106**	**0.904**
**Threonine**	4	9	2.19	**0.042**	**1.874**	**0.044**	**0.351**
**Serine**	3	7	0.29	**0.004**	**0.109**	**0.005**	**0.035**
**Valine**	5	11	4.26	**0.102**	**1.655**	**0.105**	**0.487**
**Leucine**	6	13	2.99	**0.086**	**0.896**	**0.087**	**0.398**
**Isoleucine**	6	13	2.03	**0.058**	**0.940**	**0.059**	**0.489**
**Proline**	5	9	0.86	**0.021**	**0.184**	**0.017**	**0.094**
**Aspartate**	4	7	2.44	**0.047**	**0.708**	**0.038**	**0.300**
**Glutamate**	5	9	5.29	**0.127**	**0.982**	**0.107**	**0.740**
**Phenylalanine**	9	11	4.26	**0.183**	**2.362**	**0.105**	**0.425**
**Tyrosine**	9	11	1.17	**0.050**	**1.156**	**0.029**	**3.079**
**Lysine**	6	14	1.66	**0.048**	**0.565**	**0.052**	**0.299**

Supplementary Method 2: Conditions for amino acids and isotope-labeled amino acids separation on gas chromatography-mass spectrometry (GC-MS) and gas chromatography-isotope ratio mass spectrometry (GC-IRMS)

The identity and quantity of each detectable amino acid in a sample; alanine, glycine, threonine, serine, valine, leucine, isoleucine, proline, aspartate, glutamate, phenylalanine and lysine were determined by gas chromatography-mass spectrometry (GC-MS). Asparagine and glutamine are desaminated during the acidic hydrolysis with 6 M HCl, resulting in the formation of aspartate and glutamate, respectively. Therefore, asparagine was measured together with aspartate, and glutamine with glutamate. The temperature program of the GC-MS was as follows: 70°C (5 min) to 100°C (30 min) at 30°C/min, to 175°C (5 min) at 10°C/min, to 250°C (5 min) at 10°C/min, to 340°C (15 min) at 30°C/min. The injector was set to 280°C and the helium flow to 1.7 mL/min.

The isotopic composition of each amino acid was determined using gas chromatography-isotope ratio mass spectrometry (GC-IRMS) with the following temperature program: 50°C (0 min) to 100°C (10 min) at 15°C/min, to 130°C (0 min) at 2°C/min, to 220°C (0 min) at 10°C/min, to 250°C (10 min) at 30°C/min. The injector was set to 250°C and the helium flow to 2 mL/min.

Supplementary Method 3: Derivatization correction of AAs

During derivatization of AAs additional H and C are introduced to each amino acid, thereby changing the isotope ratio of ^{13}C (at% $^{13}C/^{12}C$) as well as D (at% D/^{1}H). To account for the isotope shift during trifluoroacetylation and isopropylation of AAs, the measured isotopic enrichment was corrected according to the **Supplementary Equation 19** to yield the corrected original isotopic enrichment [31]:

$$IC_{corr} = \frac{IC_{meas} \times (n_{AA} + n_{TFAA} + n_{IP}) - IC_{TFAA} \times n_{TFAA} - IC_{IP} \times n_{IP}}{n_{AA}} \quad (19)$$

where:

n_{AA} – number of H or C in respective AA

n_{TFAA} – number of H or C of trifluoroacetic acid

n_{IP} – number of H or C of iso-propanol

IC_{corr} – corrected isotopic composition of AA

IC_{meas} – measured isotopic composition of AA

IC_{TFAA} – H or C isotopic composition of trifluoroacetic acid

IC_{IP} – H or C isotopic composition of iso-propanol.

Supplementary Method 4: Calculation of ^{13}C- and D-label integration into total NERs and tAAs

As heavy isotopes occur naturally in soil, they can mask the signal of the ^{13}C or D isotope tracer introduced with a labeled substrate. Therefore, the natural isotopic abundance amplifying the amount of ^{13}C or D derived from the isotope tracer was corrected with the help of the unlabeled substrate control. The abundance of the heavy isotope (^{13}C and D) derived from the respective labeled substrate was estimated as excess over the unlabeled controls according to **Supplementary Equation 23** for the calculation of non-extractable residues (NERs) and total amino acids (tAAs).

$$\text{Total } ^{13}C/D_{\text{blank}} = C/H_{\text{soil}} \times at\%_{\text{soil}} \quad (20)$$

$$\text{Total } ^{13}C/D_{\text{soil + unlabeled substrate}} = \text{total } ^{13}C/D_{\text{blank}} + C/H_{\text{unlabeled substrate}} \times at\%_{\text{unlabeled substrate}} \quad (21)$$

$$\text{Total } ^{13}C/D_{\text{soil + labeled substrate}} = \text{total } ^{13}C/D_{\text{blank}} + C/H_{\text{labeled substrate}} \times at\%_{\text{labeled substrate}} \quad (22)$$

$$\mathbf{^{13}C/D_{\text{excess}} = {^{13}C/D}_{\text{soil + labeled substrate}} - {^{13}C/D}_{\text{soil + unlabeled substrate}}} \quad \mathbf{(23)}$$

Where:

at%: at% $^{13}C/^{12}C$ or at% $D/^{1}H$

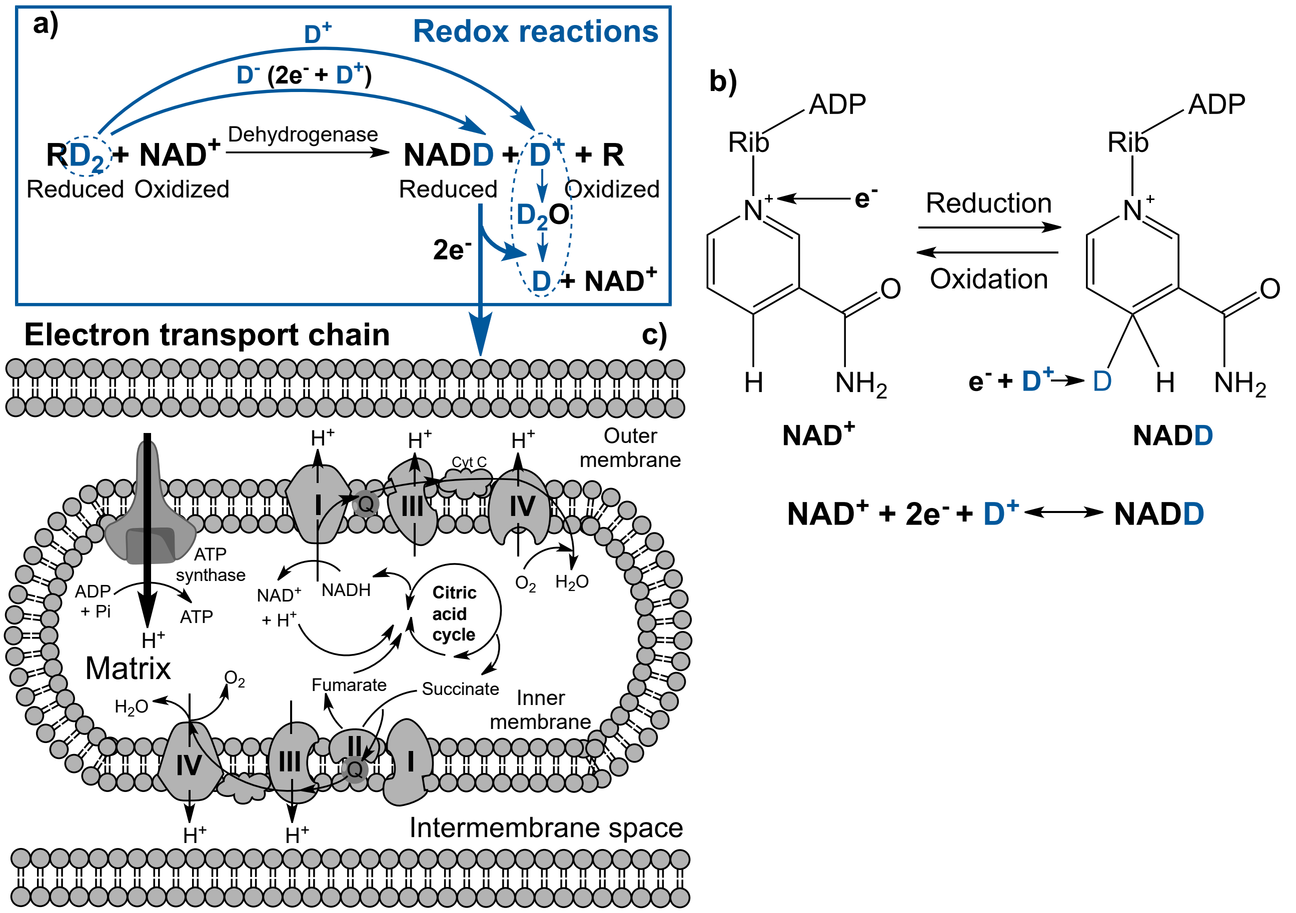

Supplementary Fig. 7: Release of D from C–D bonds of a D-labeled substrate (reactant RD_2) during its enzymatic cleavage. In energy metabolism (catabolism), a deuterated compound donates electrons in redox reactions involving the coenzyme **nicotinamide adenine dinucleotide (NAD(H)): RD_2 + NAD^+ → NADD + D^+ + R** (**a**). During the oxidation of the D-substrate (RD_2) to R, the bound deuterium is removed in the form of a hydride ion (**D^-: 2e- + D^+**) and a proton (**D^+**). The D^- is transferred to NAD forming NADD while D^+ is directly released into the ambient water solution. One e^- from the D^- electron pair is transferred by an enzyme (e.g. dehydrogenase) to the positively charged nitrogen of the nicotinamide ring of NAD^+ (**b**). The D^+ and the second e^- from the D^- are transferred to the C4 opposite this nitrogen. After the D^- is transferred to NAD^+, the NAD^+ is reduced to NADD. The $2e^-$ from the NADD are then channeled into the electron transport chain (**c**) and the D from NADD is released to water during the recycling of NADD to NAD^+. The e^- transfer is coupled with the transfer of protons across the mitochondrial membrane. This creates an electrochemical proton gradient that drives the synthesis of adenosine triphosphate (ATP), a molecule that stores energy chemically in the form of highly strained bonds.

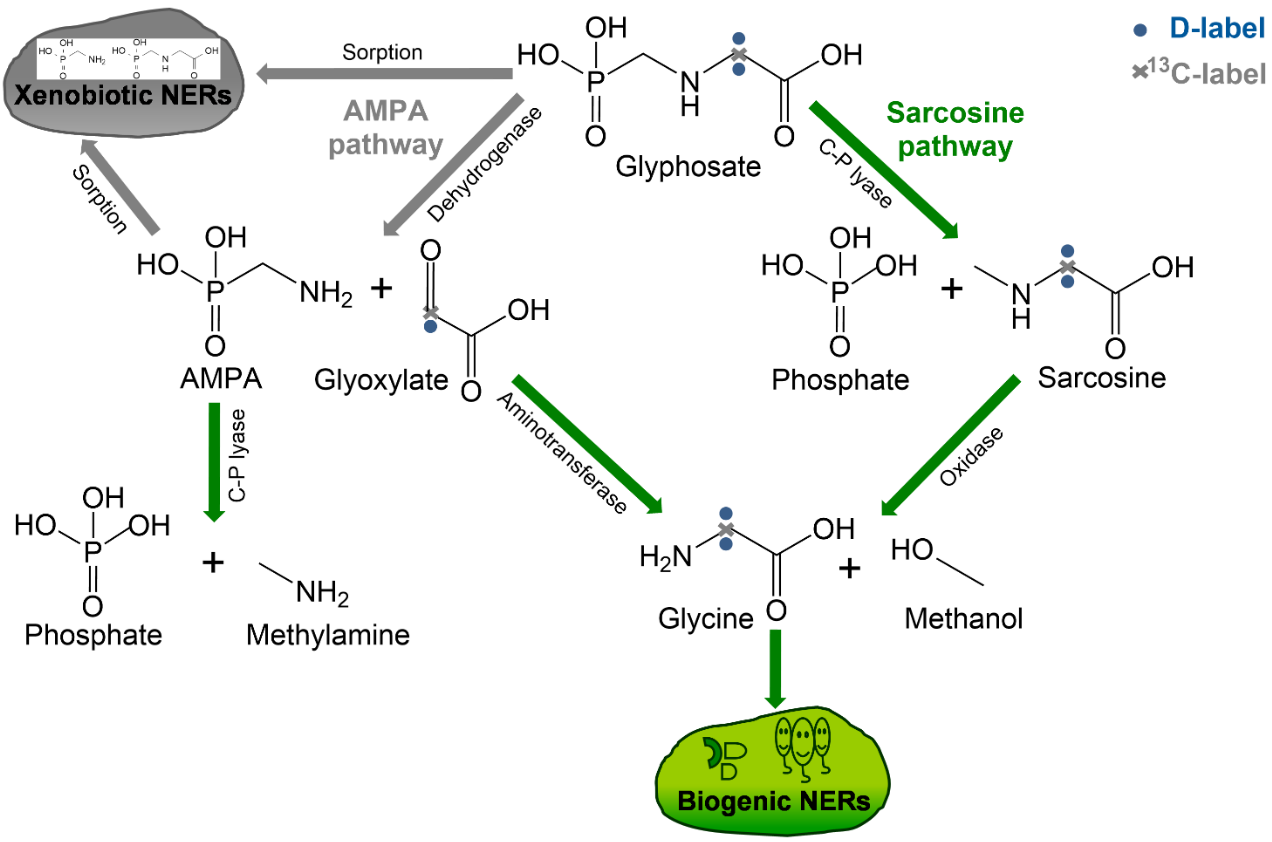

Supplementary Fig. 8: Degradation pathways of glyphosate (GLP) labeled at position 2-C leading to the formation of labeled sarcosine and glycine. Grey crosses show the labeling position of GLP with one ^{13}C, whilst the two **blue dots** indicate the two D-labels. Pathways contributing to the formation of xenobiotic non-extractable residues (xenobiotic NERs) are shown with **grey arrows**. **Green arrows** indicate pathways of biogenic non-extractable residues (biogenic NERs) formation. Text next/below the arrows (except for ‘sorption’) specifies enzymes contributing to biodegradation processes of glyphosate. In the sarcosine pathway, firstly, the C–P bond of 2-C-GLP is cleaved by the C–P lyase enzyme to form phosphate and sarcosine. Sarcosine, which retains both the ^{13}C and two D-labels from the parent molecule 2-C-GLP, is rapidly oxidized in the presence of oxidase to glycine and methanol. The double-labeled (^{13}C & two D) glycine is incorporated directly into microbial biomass which gives rise to biogenic NERs. In the presence of dehydrogenase, 2-C-GLP follows the aminomethylphosphonic acid (AMPA) pathway. For GLP labeled with ^{13}C and two D-labels at position 2, its transformation product AMPA is unlabeled, while glyoxylate retains the ^{13}C and only one D-label of the parent GLP. An NH_2-group can then be ‘added’ to glyoxylate (containing ^{13}C & one D) by aminotransferase to form glycine. The untransformed 2-C-GLP and its major transformation product AMPA can also be sorbed to soil forming xenobiotic NERs. However, AMPA may also undergo further biodegradation via C–P bond cleavage by the C–P lyase enzyme to form phosphate and methylamine, both of which will be unlabeled. Figure adapted from Nowak et al. [32].

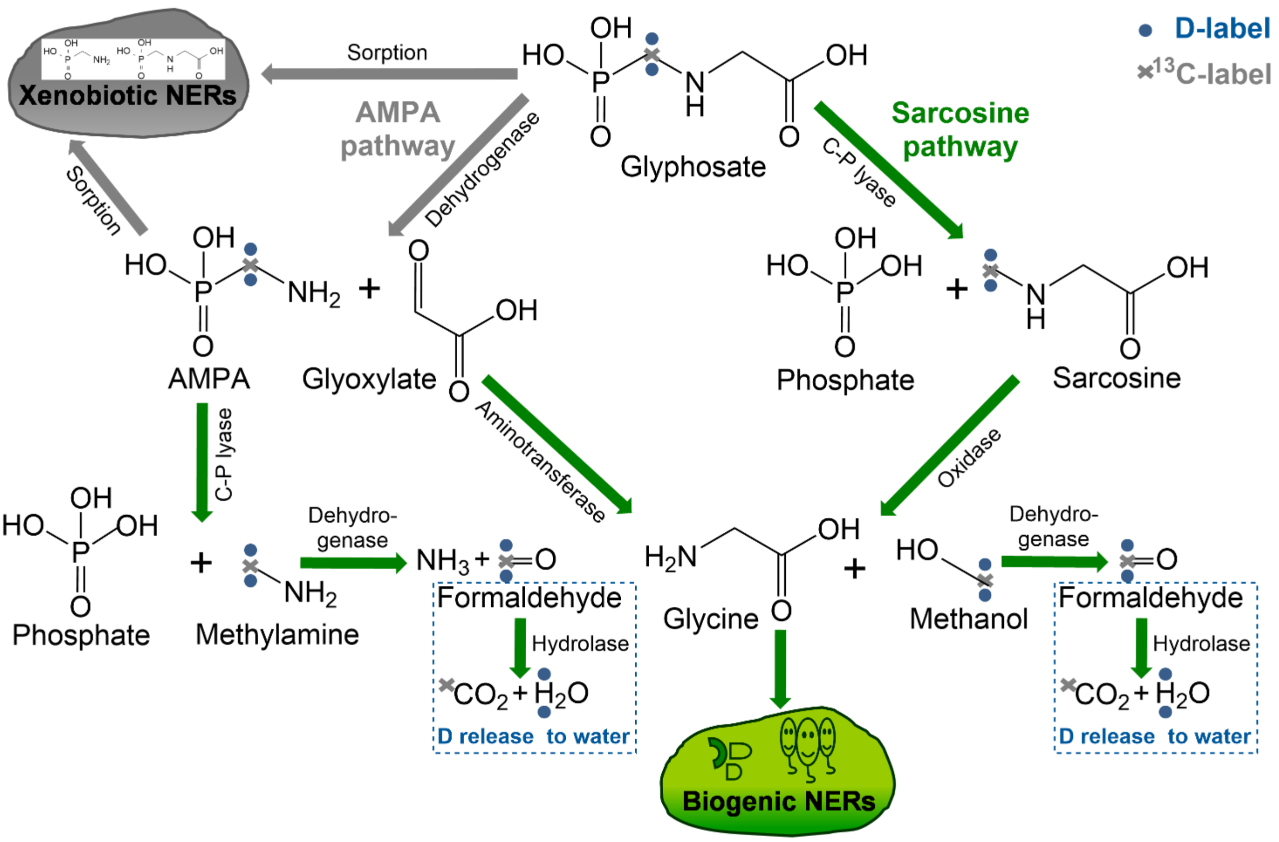

Supplementary Fig. 9: Degradation pathways of glyphosate (GLP) labeled at position 3-C leading to the formation of labeled aminomethylphosphonic acid (AMPA) and labeled sarcosine. Grey crosses show the labeling position of GLP with one ^{13}C, whilst the two **blue dots** indicate the two D-labels. Pathways contributing to the formation of xenobiotic non-extractable residues (xenobiotic NERs) are shown with **grey arrows**. **Green arrows** indicate pathways of biogenic non-extractable residue (biogenic NERs) formation. Text next/below the arrows (except for 'sorption') specifies enzymes contributing to biodegradation processes of glyphosate. **Blue dotted lines** highlight the release of D-labels from D_2-GLP labeled at position 3 to water in both degradation pathways of GLP (sarcosine and AMPA). When the C–P bond of GLP labeled at position 3 is cleaved by the C–P lyase enzyme to form phosphate and sarcosine in the sarcosine pathway, sarcosine will preserve the ^{13}C and two D-labels from GLP. However, after the oxidation of sarcosine by oxidase, the resulting glycine will lose the ^{13}C- and D-labels, whilst methanol will retain both of them. In the presence of dehydrogenase, the methanol is transformed to formaldehyde, from which the ^{13}C- and two D-labels are lost as ^{13}C-carbon dioxide and deuterated water after hydrolysis. When GLP labeled with ^{13}C and two D-labels at position 3 undergoes biodegradation via the AMPA pathway in the presence of dehydrogenase, its transformation product AMPA will preserve the labels of the parent GLP, while glyoxylate will lose both labels. Therefore, in both biodegradation pathways (sarcosine & AMPA), unlabeled glycine will be formed and incorporated into microbial biomass and ultimately biogenic NERs. The untransformed 3-C-GLP and its major transformation product AMPA can also be sorbed to soil forming xenobiotic NERs. If the C–P bond of AMPA containing ^{13}C and two Ds is cleaved by the C–P lyase enzyme to form phosphate and methylamine, the methylamine will preserve both labels. The ^{13}C- and both D-labels are then ultimately released to ^{13}C-labeled carbon dioxide and deuterated water. Figure adapted from Nowak et al. [32].

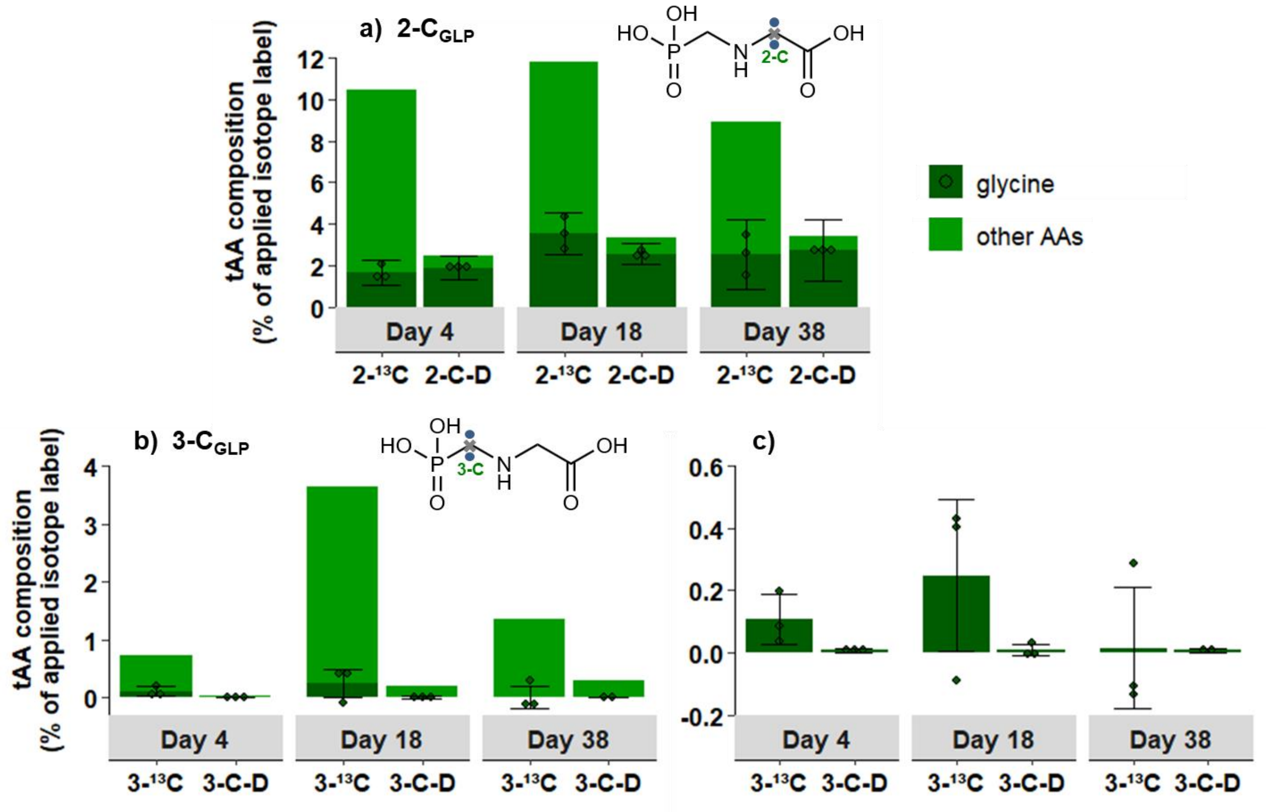

Supplementary Fig. 10: Contribution of the amino acid glycine to the total measured amino acid (tAA) pool for glyphosate (GLP) labeled at position 2 (a) or position 3 (b) with ^{13}C or D. Panel (**c**) shows figure (**b**) with enlarged scale for a better visibility of small values. Bars represent the average of triplicate measurements (n = 3, except 3-C–$D_{2\text{-GLP}}$ on day 38, where n=2) and error bars the propagated standard deviation. Dots show individual replicates, which resulted in negative values in case they were below the background abundance in the unlabeled treatment. The differences between $^{13}C_{glycine}$ and $D_{glycine}$ contents were not statistically significant on any sampling day for 2-C_{GLP} or 3-C_{GLP}. The GLP structures above (**a** and **b**) indicate the isotope label positions with **grey cross**: ^{13}C-label, and two **blue dots**: D-labels. For GLP labeled at position 2, the contents of glycine were comparable for both isotopes on all sampling dates and this amino acid dominated the pool of tAAs **(a)**. In contrast, for GLP labeled at position 3, glycine did not predominate in the tAA pool (**b**, **c**).

Supplementary References

1. Trapp, S., Brock, A. L., Nowak, K. & Kästner, M. Prediction of the Formation of Biogenic Nonextractable Residues during Degradation of Environmental Chemicals from Biomass Yields. *Environmental science & technology* **52,** 663–672; 10.1021/acs.est.7b04275 (2018).

2. Brock, A. L. *et al.* Microbial Turnover of Glyphosate to Biomass: Utilization as Nutrient Source and Formation of AMPA and Biogenic NER in an OECD 308 Test. *Environmental science & technology* **53,** 5838–5847; 10.1021/acs.est.9b01259 (2019).

3. Berry, D. *et al.* Tracking heavy water (D_2O) incorporation for identifying and sorting active microbial cells. *Proceedings of the National Academy of Sciences of the United States of America* **112,** E194-203; 10.1073/pnas.1420406112 (2015).

4. Nowak, K. M., Miltner, A., Gehre, M., Schäffer, A. & Kästner, M. Formation and fate of bound residues from microbial biomass during 2,4-D degradation in soil. *Environmental science & technology* **45,** 999–1006; 10.1021/es103097f (2011).

5. Muskus, A. M., Krauss, M., Miltner, A., Hamer, U. & Nowak, K. M. Effect of temperature, pH and total organic carbon variations on microbial turnover of $^{13}C_3{}^{15}N$-glyphosate in agricultural soil. *The Science of the total environment* **658,** 697–707; 10.1016/j.scitotenv.2018.12.195 (2019).

6. Girardi, C. *et al.* Microbial degradation of the pharmaceutical ibuprofen and the herbicide 2,4-D in water and soil - use and limits of data obtained from aqueous systems for predicting their fate in soil. *The Science of the total environment* **444,** 32–42; 10.1016/j.scitotenv.2012.11.051 (2013).

7. Padilla, J. T. & Selim, H. M. Time-Dependent Sorption and Desorption of Glyphosate in Soils: Multi-reaction Modeling. *Vadose Zone Journal* **18,** 1–10; 10.2136/vzj2018.12.0214 (2019).

8. Gros, M., Rodríguez-Mozaz, S. & Barceló, D. Rapid analysis of multiclass antibiotic residues and some of their metabolites in hospital, urban wastewater and river water by ultra-high-performance liquid chromatography coupled to quadrupole-linear ion trap tandem mass spectrometry. *Journal of chromatography. A* **1292,** 173–188; 10.1016/j.chroma.2012.12.072 (2013).

9. Archundia, D. *et al.* Assessment of the Sulfamethoxazole mobility in natural soils and of the risk of contamination of water resources at the catchment scale. *Environment International* **130,** 104905; 10.1016/j.envint.2019.104905 (2019).

10. Kurwadkar, S. T., Adams, C. D., Meyer, M. T. & Kolpin, D. W. Comparative mobility of sulfonamides and bromide tracer in three soils. *Journal of environmental management* **92,** 1874–1881; 10.1016/j.jenvman.2011.03.018 (2011).

11. Winter, J. de. Using the Student's t-test with extremely small sample sizes, 2013.

12. Fox, J. & Weisberg, S. *An {R} Companion to Applied Regression*. Available at https://socialsciences.mcmaster.ca/jfox/Books/Companion/ (Sage, Thousand Oaks {CA}, 2019).

13. Dinno, A. *conover.test: Conover-Iman Test of Multiple Comparisons Using Rank Sums.* R package version 1.1.5. Available at https://CRAN.R-project.org/package=conover.test (2017).

14. Mangiafico, S. S. *rcompanion: Functions to Support Extension Education Program Evaluation.* version 2.4.30. Available at https://CRAN.R-project.org/package=rcompanion (2023).

15. Kassambara, A. *ggpubr: 'ggplot2' Based Publication Ready Plots.* R package version 0.5.0. Available at https://CRAN.R-project.org/package=ggpubr (2022).

16. Wickham, H. *ggplot2: Elegant Graphics for Data Analysis*. Available at https://ggplot2.tidyverse.org (Springer-Verlag New York, 2016).

17. Brock, A. L., Kästner, M. & Trapp, S. Microbial growth yield estimates from thermodynamics and its importance for degradation of pesticides and formation of biogenic non-extractable residues. *SAR and QSAR in Environmental Research* **28,** 629–650; 10.1080/1062936X.2017.1365762 (2017).

18. Trapp, S., Brock, A. L., Kästner, M., Schäffer, A. & Hennecke, D. Critical evaluation of the microbial turnover to biomass approach for the estimation of biogenic non-extractable residues (NER). *Environmental sciences Europe* **34,** 15; 10.1186/s12302-022-00592-5 (2022).

19. Wu, X. *et al.* Maize (Zea mays L.) Plants Alter the Fate and Accumulate Nonextractable Residues of Sulfamethoxazole in Farmland Soil. *Environmental science & technology* **58,** 9292–9302; 10.1021/acs.est.3c08954 (2024).

20. Wang, S. *et al.* (Bio)degradation of glyphosate in water-sediment microcosms - A stable isotope co-labeling approach. *Water research* **99,** 91–100; 10.1016/j.watres.2016.04.041 (2016).

21. Dadfar, E. & Shafiei, F. Prediction of some thermodynamic properties of sulfonamide drugs using genetic algorithm-multiple linear regressions. *J Chin Chem Soc* **67,** 492–513; 10.1002/jccs.201900232 (2020).

22. Dean, J. A. *Lange's Handbook of Chemistry. 12th ed.* (McGraw-Hill, New York, 1979).

23. Busch, R. *et al.* Measurement of protein turnover rates by heavy water labeling of nonessential amino acids. *Biochimica et biophysica acta* **1760 5,** 730–744 (2006).

24. Frederique, E. *et al.* An updated review on tritium in the environment. *Journal of environmental radioactivity* **181,** 128–137 (2018).

25. Paul, A. *et al.* Hydrogen dynamics in soil organic matter as determined by 13 C and 2 H labeling experiments. *Biogeosciences* **13,** 6587–6598 (2016).

26. Kostyukevich, Y. *et al.* Hydrogen/deuterium exchange in mass spectrometry. *Mass spectrometry reviews* **37,** 811–853; 10.1002/mas.21565 (2018).

27. Liu, X.-J. A. *et al.* The soil priming effect: Consistent across ecosystems, elusive mechanisms. *Soil Biology and Biochemistry* **140,** 107617; 10.1016/j.soilbio.2019.107617 (2020).

28. DIN. *DIN 32645:2008-11: Chemical Analysis - Decision limit, detection limit and determination limit under repeatability conditions - Terms, methods, evaluation.* (Deutsches Institut für Normung, Berlin, Germany, 2008).

29. Hofstetter, T. B. *et al.* Perspectives of compound-specific isotope analysis of organic contaminants for assessing environmental fate and managing chemical pollution. *Nat Water* **2,** 14–30; 10.1038/s44221-023-00176-4 (2024).

30. *The fitness for purpose of analytical methods. A laboratory guide to method validation and related topics.* 2nd ed. (Eurachem, Teddington, 2014).

31. Silfer, J. A., Qian, Y., Macko, S. A. & Engel, M. H. Stable carbon isotope compositions of individual amino acid enantiomers in mollusc shell by GC/C/IRMS. *Organic Geochemistry* **21,** 603–609; 10.1016/0146-6380(94)90006-X (1994).

32. Nowak, K. M., Miltner, A. & Kästner, M. Environmental Fate Assessment of Chemicals and the Formation of Biogenic Non-extractable Residues (bioNER). In *Bioavailability of Organic Chemicals in Soil and Sediment*, edited by J. J. Ortega-Calvo & J. R. Parsons (Springer International Publishing, Cham, 2020), pp. 81–111.
